# AIE-active two-photon fluorescent nanoprobe with NIR-II light excitability for highly efficient deep brain vasculature imaging

**DOI:** 10.7150/thno.53780

**Published:** 2021-01-01

**Authors:** Soham Samanta, Meina Huang, Shaoqiang Li, Zhigang Yang, Ying He, Zhenyu Gu, Jianguo Zhang, Dan Zhang, Liwei Liu, Junle Qu

**Affiliations:** Center for Biomedical Photonics & Key Laboratory of Optoelectronic Devices and Systems of Guangdong Province and Ministry of Education, College of Physics and Optoelectronic Engineering, Shenzhen University, Shenzhen 518060, China.

**Keywords:** aggregation-induced emission (AIE), two-photon imaging, second near-infrared (NIR-II) excitation, brain Imaging, AIE nanoparticle

## Abstract

Aggregation induced emission (AIE)-active bright two-photon fluorescent probes with second near-infrared (NIR-II) light excitability can be used for efficient brain bioimaging studies, wherein the fabrication of water-dispersible nanoparticles by encapsulating the hydrophobic probes with amphiphilic polymer holds the key to ensuring biocompatibility and *in vivo* adaptability. However, barely any study has evaluated the structural requirements that can substantially affect the water-dispersible nanoparticle formation ability of an organic AIE-active dye with amphiphilic polymers. The present study systematically assessed the structural dependency of a well-known acrylonitrile based AIE system/fluorogenic core upon the formation of water-dispersible nanoparticles and elucidated how the structural modifications can impact the *in vivo* two-photon imaging.

**Methods:** A total of four acrylonitrile-based aggregation induced emission (AIE)-active two-photon (TP) fluorescent probes (AIETP, AIETP C1, AIETP C2 and AIETP C3) have been judiciously designed and synthesized with structural variations to realize how the structural alterations could substantially influence the water-dispersible nanoparticle formation ability (with amphiphilic polymers) and photo-stability to impact the *in vivo* imaging.

**Results:** It has been found that the incorporation of the phenyl-thiazole unit in AIETP, AIETP C2 and AIETP C3 facilitated the formation of water-dispersible nanoparticles (NPs) with amphiphilic polymers (Pluronic F127) whereas the presence of only phenyl moiety instead in AIETP C1 could not meet the suitable condition to form the NPs with good aqueous dispersibility. Rationally designed AIETP NPs that exhibited higher brightness, improved photostability and good two-photon absorption cross section was successfully employed for *in vivo* brain vasculature imaging.

**Conclusions:** Robust noninvasive 2D and 3D two-photon (NIR-II light, 1040 nm) brain vasculature imaging with beneficial attributes such as outstanding penetration depth (800 µm) and exceptional spatial resolution (1.92 µm), were achieved by utilizing AIETP NPs in this study.

## Introduction

In neuroscience related biomedical research, high resolution deep tissue imaging has sparked immense interest because of the increasing necessities to visualize the live dynamic changes 1-3. Even though magnetic resonance imaging (MRI) and X-ray computed tomography (CT) are mainly used for deep-penetration brain imaging in clinical diagnosis, widespread utilization of these methods is very limited owing to the high cost and low spatial resolution. In this regard, two-photon fluorescence microscopy (TPM) has come across as a superior choice to realize higher resolution *in vivo* deep tissue imaging than the usual one photon microscopy (OPM) 4-9. Since, a fluorophore with visible absorption/excitation can be excited by low energy NIR light in TPM setup, it can substantially reduce the extent of photo-bleaching and facilitate the better imaging penetration depth by lowering the biological tissue induced light attenuation 6, 10-13. In addition, the nonlinear optical process in TPM can adhere to the advantages of inherent 3D imaging because of the square dependence of two-photon fluorescence on the incident light intensity. Therefore, designing an efficient two-photon fluorescent probe for effective deep tissue brain imaging is an urgent need.

In comparison with the traditional femtosecond (fs) near-infrared (NIR-I) (700-1000 nm) excitation laser, the NIR-II (1000-1700 nm) excitation laser with reduced photon scattering can more effectively penetrate the biological tissues and focus more precisely on the area of interest beneath biological tissues to provide improved deep-tissue imaging practice 14-19. An NIR-II biological window excitable two-photon fluorescent probe is always advantageous over NIR-I excitable counterpart 20-23. However, till now very few two-photon probes with NIR-II excitation have been reported.

Tackling the aggregation-caused quenching (ACQ) effect of traditional small molecule organic probes during their use in two-photon imaging application is another important aspect that can hamper the imaging efficacy. Aggregation induced emission (AIE) active two-photon probes have been overwhelmingly developed recently to address these concerns 24-27. NIR-II light excitable AIE-active two-photon fluorescent probes can be considered as the preferred choice for deep-penetration brain imaging. Acrylonitriles with aggregation-induced emission (AIE) characteristics have been found to show promising applications in this context. Rationally designed acrylonitriles that can reveal AIE features and nonlinear optical characteristics are expedient for efficient two-photon excited imaging in live samples 27. The flexibility in choosing suitable conjugated donor and acceptor parts to improve the two-photon cross section and tuning π-conjugation to shift the excitation/emission in red/NIR region has made acrylonitriles an interesting building unit for two-photon imaging 6, 27.

However, biocompatibility and aqueous solubility remained as the major challenges in the utilization of two-photon acrylonitrile based AIE probes in imaging practices. Attempts have been made by attaching hydrophilic groups to the organic fluorophores to improve aqueous solubility. However, such modifications often compromise the fluorescence intensities 28, 29. Consequently, encapsulation of the organic fluorophore with amphiphilic polymers to form aqueous dispersible nanoparticle has become an attractive strategy to employ these probes in bio-imaging 30. But synthesizing uniform organic nanoparticles is challenging and the nanoparticle formation aptitude of a dye with amphiphilic polymers may vary substantially from probe to probe. Till now, hardly any attempt has been made to find out the structural requirements that can substantially alter the water-dispersible nanoparticle formation ability of an organic AIE-active dye with amphiphilic polymers.

**Scheme 1:**
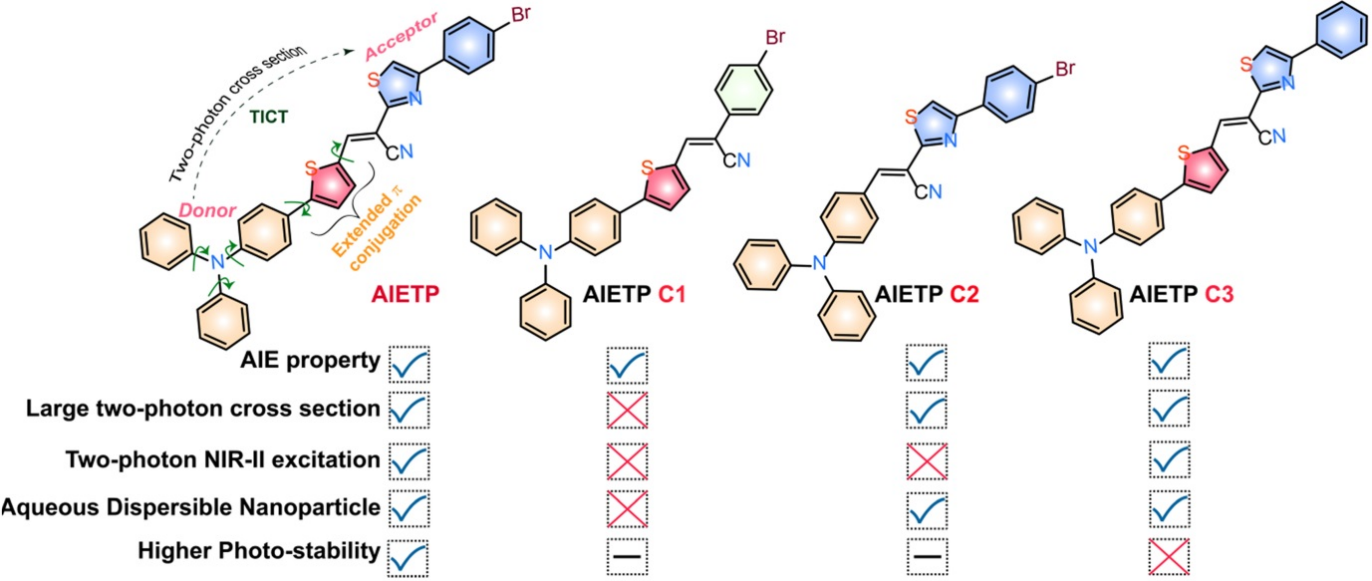
Rational design of the NIR-II excitable AIE-active two-photon fluorescent probes for *in vivo* brain imaging.

In this context, herein, we have presented the structural outlook to elucidate how the structural modifications in an acrylonitrile based AIE system/fluorogenic core could substantially influence the water-dispersible nanoparticle formation ability (with amphiphilic polymers such as Pluronic F127) and impact the *in vivo* two-photon imaging. Pluronic F-127 (F127) has been preferentially chosen as the polymeric matrix, for the encapsulation of the AIE-gens to form the water-dispersible NPs as it is a widely used US Food and Drug Administration (FDA) approved surfactant polymer 31-32. An AIE-active two-photon fluorescent probe AIETP was judiciously designed along with three other control compounds (AIETP C1, AIETP C2 and AIETP C3) to study the influence of structural amendments in specific bioimaging applications (Scheme 1 and Scheme S1, Supporting Information). AIETP with excellent water-dispersible nanoparticle formation ability and two-photon NIR-II light excitability demonstrated higher brightness, improved photostability and better two-photon absorption cross section among all the four designed probes which was successfully utilized for efficient brain vasculature imaging.

## Experimental Methods

### General information and materials

For the synthesis of fluorescent probes, all the starting materials were purchased from commercial suppliers and used without further purification. Other chemicals for biological experiments and analytical studies (such as phosphate buffered saline (1×, pH: 7.4), 2',7'-dichlorofluorescin diacetate (DCFDA), Rhodamine B etc.) were also bought from authentic commercial suppliers. To record the absorption spectra, a Cintra 2020 UV-Vis spectrophotometer was used and the spectral data were recorded in the range of 300-800 nm wavelength using quartz cuvettes with 10 mm path length at 298 K. Similarly, the fluorescence measurements were performed on a Horiba Fluoromax-4 spectrofluorometer using 10 mm path length quartz cuvettes with a slit width of 3 nm at 298 K. Dynamic light scattering (DLS) studies were conducted in a Brookhaven Instrument to analyse the particle size. Transmission electron microscopy (TEM) analysis was performed with a F200 TEM instrument. The mass spectra of the fluorescent probes were obtained using ThermoScientific Q Exactive mass spectrometer and the mass samples of the probes were prepared in acetonitrile. A drop of the diluted trifluoroacetic acid (TFA) solution was added to each sample for ionization before acquiring the mass spectra since the probes are uncharged. The Nuclear magnetic resonance (NMR) spectra were recorded on a 400 MHz BRUKER AVANCE 300 instrument. The chemical shifts were recorded in parts per million (ppm) on the scale. The spin multiplicities in ^1^H NMR spectra are described using the following abbreviations: s = singlet; d = doublet; t = triplet; q = quartet; m = multiplet.

### Synthesis and characterization of the fluorescent probes

#### AIETP

At first, 5-(4-(diphenylamino)phenyl)thiophene-2-carbaldehyde was synthesized by the Suzuki coupling reaction 33. 11 mmol (1.71 g) of (5-formylthiophen-2-yl) boronic acid and the 10 mmol (3.24 g) of 4-Bromotriphenylamine compound were dissolved in 35 mL of dioxane in a round bottom flask. Subsequently, 0.55 mmol of Pd(PPh_3_)_4_ and 20 mmol of Na_2_CO_3_ (aqueous solution) were added to it under a nitrogen atmosphere and the reaction mixture was refluxed for 24h with continuous stirring. A dark black reaction mixture was obtained which was filtered after it cooled down to room temperature. To the filtrate, DCM (50 mL) and water (30 mL) were added and the organic layer was separated using separating funnel. It was dried over the MgSO_4_ and the solvent was then evaporated to get the crude product. The final pure yellow solid product of 5-(4-(diphenylamino)phenyl)thiophene-2-carbaldehyde was then obtained from the column chromatography (eluent EtOAc/Hexane in the ratio of 5:95) (Scheme S1, 1st step). In the next step, 2.0 mmol of 5-(4-(diphenylamino)phenyl)thiophene-2-carbaldehyde and 2.0 mmol of 4-(4-Bromophenyl)-2-thiazoleacetonitrile were dissolved in EtOH. The resultant mixture was then refluxed in presence of 3.0 mmol NaOAc for 12 h to yield the brown-red product of AIETP (Scheme S1). The product was filtered, washed with methanol several times and dried in open air.

Calculated yield (for the final step): 86%. ^1^H NMR [400 MHz, DMSO-d_6_, TMS, *J* (Hz), δ (ppm)]: 8.52 (1H, s), 8.30 (1H, s), 7.99 (2H, d, *J* = 6.8), 7.94 (1H, d, *J* = 3.2), 7.69-7.67 (4H, m), 7.62 (1H, d, *J* = 3.2), 7.37 (4H, t, *J* = 6.4), 7.14 (2H, d, *J* = 6.0), 7.11 (4H, d, *J* = 6.4), 6.99 (2H, d, *J* = 6.8). ^13^C NMR [100 MHz, CDCl_3_, TMS, δ (ppm)]:162.9, 155.7, 152.2, 149.0, 147.1, 137.0, 136.8, 135.1, 132.9, 132.1, 129.6, 128.2, 127.7, 127.3, 126.4, 125.2, 123.9, 123.2, 122.7, 122.6, 113.9, 99.8. ESI-MS (positive mode, m/z) Calculated for C_34_H_23_BrN_3_S_2_: 616.0512, 618.0491. Found: 618.0487 [(M + H^+^)].

#### AIETP C1

First, 5-(4-(diphenylamino)phenyl)thiophene-2-carbaldehyde was synthesized following the same procedure mentioned in the detailed synthetic procedure of AIETP. In the final step, 1.0 mmol of 5-(4-(diphenylamino)phenyl)thiophene-2-carbaldehyde and 1.0 mmol of 2-(4-bromophenyl)acetonitrile were refluxed in EtOH in presence of 1.5 mmol NaOAc for 10 h to yield the orange solid product of the control compound AIETP C1 which was then filtered, washed several times with cold methanol and dried in open air (Scheme S1).

Calculated yield (for the final step):79%. ^1^H NMR [400 MHz, CDCl_3_, *J* (Hz), δ (ppm)]: 7.59 (1H, s), 7.57-7.49 (6H, m), 7.57-7.49 (7H, m), 7.57-7.49 (7H, m). ESI-MS (positive mode, m/z) Calculated for C_31_H_22_BrN_2_S: 533.0682, 535.0662. Found: 533.0682, 535.0658 [(M + H^+^)].

#### AIETP C2

In a round bottom flask, 1.0 mmol of 4-(diphenylamino)benzaldehyde and 1.0 mmol of 4-(4-Bromophenyl)-2-thiazoleacetonitrile were dissolved in dry EtOH. The resultant mixture was refluxed in presence of 1.5 mmol NaOAc for 17 h to yield the orange-yellow solid product of AIETP C2 (Scheme S1). The reaction mixture was allowed to come to the room temperature and the precipitate was filtered. The orange-yellow solid product was washed with cold methanol several times and dried in open air.

Calculated yield (for the final step): 88%. ^1^H NMR [400 MHz, CDCl_3_, *J* (Hz), δ (ppm)]: 8.10 (1H, s), 7.86 (4H, t, *J* = 8.0), 7.57 (2H, d, *J* = 8.4), 7.49 (1H, s), 7.36 (4H, dd, *J* = 8.4), 7.19-7.15 (6H, m), 7.04 (2H, d, *J* = 8.8). ^13^C NMR [100 MHz, CDCl_3_, TMS, δ (ppm)]:163.9, 156.5, 151.3, 146.2, 144.1, 133.0, 132.0, 131.9, 129.8, 129.7, 128.2, 126.3, 125.1, 124.8, 122.6, 120.1, 117.8, 113.5. ESI-MS (positive mode, m/z) Calculated for C_30_H_21_BrN_3_S: 534.0635, 536.0614. Found: 534.0634 [(M + H^+^)].

#### AIETP C3

1.0 mmol of 5-(4-(diphenylamino)phenyl)thiophene-2-carbaldehyde (synthesized following first step of Scheme S1) and 1.0 mmol of 4-phenyl-2-thiazoleacetonitrile were dissolved in dry EtOH and the resultant mixture was refluxed with continuous stirring in presence of NaOAc for 20 h to yield the orange-red product of the control compound AIETP C3. The solid product was filtered after the reaction mixture came to the room temperature (Scheme S1). It was then washed thoroughly with methanol and dried in open air.

Calculated yield (for the final step): 53%. ^1^H NMR [400 MHz, CDCl_3_, J (Hz), δ (ppm)]: 8.34 (1H, s), 7.97 (2H, d, *J* = 7.2), 7.68 (1H, d, *J* = 4.4), 7.55-7.50 (3H, m), 7.47 (2H, t, *J* = 7.2), 7.39 (1H, t, *J* = 7.2), 7.32-7.28 (5H, m), 7.15-7.05 (8H, m).^ 13^C NMR [100 MHz, CDCl_3_, TMS, δ (ppm)]:162.6, 156.9, 151.9, 148.9, 147.1, 136.9, 136.6, 135.2, 134.0, 129.6, 128.9, 128.7, 127.2, 126.6, 126.4, 125.2, 123.9, 123.1, 122.6, 117.5, 113.6, 99.9. ESI-MS (positive mode, m/z) Calculated for C_34_H_24_N_3_S: 538.1407. Found: 538.1404 [(M + H^+^)].

### Fabrication of nanoparticles

The well-documented nanoprecipitation technique with slight modifications was followed to synthesize the nanoparticles for the AIE probes. Briefly, in each of the four different vials 1 mg of AIETP or AIETP C1 or AIETP C2 or AIETP C3 was dissolved in 1 mL of CHCl_3_ separately. Similarly, in each of the 4 different vials 10 mg of Pluronic F127 was dissolved in 1 mL CHCl_3_ separately. Subsequently, each of the probe solutions was mixed with each of the Pluronic F127 solutions and sonicated for 10 min to prepare a homogeneous solution. The CHCl_3_ was then fully evaporated from each of the four mixtures carefully using the rotary vapour instrument. 1 mL of water was added to each of the samples and all the 4 samples were then sonicated again for 10 min to dissolve the solid residues. For AIETP, AIETP C2 and AIETP C3 the solid residues dissolved/dispersed frequently in aqueous solutions whereas that of the AIETP C1 remained very poorly dispersible (Scheme S2, Supporting Information). The solutions were then filtered with 0.45 µm filter and used as nanoparticles (AIETP NPs, AIETP C1 NPs*, AIETP C2 NPs and AIETP C3 NPs) of the corresponding probes for studies. (*Due to very poor solubility, filtration with 0.45 µm filter resulted in the aqueous solution of AIETP C1 NPs* with negligible probe concentration).

### Fluorescence quantum yield measurement

The relative fluorescence quantum yield (η) of AIETP NPs in aqueous media was calculated using freshly prepared Rhodamine B as the standard (η = 0.7 in methanol). The η value of AIETP NPs was found to be 0.06 when calculated using the following equation:


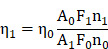


where, A is the absorbance at the excitation wavelength, F represents the integrated fluorescence intensity, n is the refractive index of the used solvent, and the subscripts 1 and 0 signify the experimental sample and the standard respectively.

### Two-photon properties and the two-photon cross section

The aqueous solution of AIETP NPs, AIETP C2 NPs, AIETP C3 NPs and the AIETP C1 NPs* (*very poor solubility in water) were loaded in cell dishes and excited using femtosecond laser at different wavelengths from 820 to 1200 nm. The average two-photon emission intensities were calculated by Image J software for the four different samples.

To calculate the two-photon absorption cross section (σ_2_) value of the NPs, following equation was used:


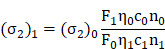


where F represents the two-photon emission intensity, η signifies the fluorescence quantum yield, c is the molar concentration of probe, n denotes the refractive index of the solvent, and the subscripts 1 and 0 represent the probe AIETP NPs and reference Rhodamine B, respectively.

### Cytotoxicity and cell imaging studies of AIETP NPs

Cytotoxicity studies were performed with the HeLa cells to evaluate the cell viability using Cell Counting Kit-8 (CCK-8) assay. Essentially, prior to CCK8 assay, 10^4^ cells per well were seeded into a 96-well tissue culture plate and incubated in a humidified incubator for adherence. After 24 h of culturing, the medium in each well was replaced by 100 μL of fresh Roswell Park Memorial Institute medium (RPMI-1640) containing varying concentrations (0-150 μM) of AIETP NPs and incubated for a period of 24 h. The solution was filtrated subsequently using 0.22 μm sterile filter. The CCK-8 reagent, diluted by RPMI-1640 medium (10%) was added to each well after the removal of culture media and the plate was incubated for 0.5 h at 37°C. Later, the absorbance was measured at 450 nm on a plate reader (RT-6100, Rayto Life and Analytical Sciences Co., Ltd, Shenzhen, China). Each trial was performed with six parallel wells. Cell viability rate was determined using the formula VR = (As-Ab)/(Ac-Ab) × 100%, where As is the absorbance of the experimental group, Ac is the absorbance of the control group (no AIETP NPs), and Ab is the absorbance of the blank group (only CCK-8).

Cell imaging studies were performed with the HeLa cancer cells which were cultured in the RPMI-1640 medium. The culturing medium was supplemented with 10% (v/v) Fetal Bovine Serum FBS (gibco) and 1 % antibiotics (10000 Units/mL penicillin and 10000 µg/mL streptomycin, gibco) under 5% CO_2_ atmosphere and 95% humidity at 37 °C until the HeLa cells became approximately 80% confluent. The cells were then utilized for the imaging studies. The single photon and two photon cell imaging studies were then performed in a Nikon A1 Confocal Microscope. The cells were incubated with AIETP NPs in the culture medium for 24 h, followed by the removal of the medium and triple washing with phosphate buffered saline (PBS) before acquiring the cell imaging.

### Intracellular ROS evaluation

Following the same procedure mentioned in the previous section HeLa cells were cultured until the cells were approximately 80% confluent. The cells were then incubated with AIETP NPs (5 µM) for 24 h. The cells were then washed with 1×PBS and incubated with DCFDA (20 µM) for 30 min. Again, the cells were washed with 1×PBS and subjected to 1040 nm two-photon light (8 mW) irradiation for 120 scans (a total of 12.68 min). Eventually, the cell imaging studies were performed with these cells with the Nikon A1 Confocal Microscope. Fluorescence emissions for DCFDA were collected in the range of 500-550 nm while the excitation was provided at 488 nm. On the other hand, 1040 nm two-photon excitation was used for AIETP NPs and the emission was acquired at the range of 604-679 nm.

### *In vivo* brain imaging studies

The animals, used for the *in vivo* studies were treated according to the protocols and the guidelines approved by Ethics committee of experimental animals, Medical department, Shenzhen University. The animals were prepared for the experiments following the standard procedure. Balb/c mice (male, 7-9 weeks old), provided by Guangdong Medical Laboratory Animal Centre were subjected to the *in vivo* two-photon fluorescence imaging experiment. In brief, the mouse was anesthetized first with a gaseous anaesthesia system (R500IP, RWD). A heating blanket was utilized to maintain the body temperature of mouse at 36 °C, and the head hair was removed and skin incision was performed to expose the skull. Thereafter, the head of a mouse was immobilized on a stereotactic stage with a heating blanket, a home-made titanium alloy piece with threaded holes was glued to the skull using dental cement. A small piece of skull bone with diameter around 3 mm was excised by a dental drill. The visual exposed mouse brain image under the experimental set up has been depicted in Scheme S3 before it was subjected to the *in vivo* imaging study. The aqueous solution (200 µL) of the prepared AIETP NPs (4.35×10^-9^ M) was administered through retro-orbital injection into the mouse vein. Femtosecond laser was focused on the surface of the brain through a 20× water immersion objective and z-stack imaging was performed with 5 μm step. The *in vivo* two-photon fluorescence imaging studies were then performed by setting the two-photon excitation laser at 1040 nm (NIR-II) wavelength.

### Selecting the area for *in vivo* brain imaging

Since the positions of region 1 and 2 of the skull (Scheme S4, Supporting Information) are too small to meet the requirements of the cranial window of 3 mm; region 3 or 4 can only be chosen for imaging experiment. Before removing the skull bone, few drops of saline were poured onto the brain to make it translucent, so that the distribution of blood vessels on region 3 and 4 can be observed. Region 3, with fewer thick blood vessels, was finally selected for the extensive *in vivo* brain imaging studies, since the thick blood vessels in region 4 might interfere with the signals from the tiny deep blood vessels (Scheme S5, Supporting Information). Craniotomies were performed centered at 2 mm posterior and 2 mm lateral to the bregma point.

However, both the region 3 and 4 can be imaged using AIETP NPs, which was confirmed by *in vivo* imaging experiment (Scheme S4, Supporting Information) confirming the whole brain labelling aptitude of AIETP NPs.

### *In vivo* toxicity

For *in vivo* histological examination, Balb/c mice were intravenously injected with 200 μL of AIETP NPs (1 mg·mL^-1^ in 1×PBS), and major organs including brain, kidney, lung, spleen, liver, and heart were collected and stained with hematoxylin and eosin (H&E) after 6 days of administration. The control group was treated with only 200 μL of 1× PBS. The images were taken by an upright microscope (Leica EG11504) with a 200× objective.

## Results and Discussion

### Rational design, synthesis and photophysical characterizations of the AIE fluorescent probes

A two-photon probe with reasonable two-photon cross section should consist of a well-organized donor-acceptor π-system 6, 27. However, the AIE phenomenon as well as the intensity of the probes can be negatively affected by the presence of intramolecular charge transfer (ICT) feature. In this context, balancing the two in a structural moiety is highly important to develop an AIE probe with efficient two-photon imaging credentials. Hence, we have incorporated a known donor moiety triphenylamine (TPE) in the probe design which not only comprises the components with free rotation to ensure the presence of AIE features but also facilitates the twisted intramolecular charge transfer (TICT) process. A total of four TPE based acrylonitriles have been synthesized (Scheme S1, Supporting Information) with structural variations as depicted in Scheme 1.

For AIETP, AIETP C1 and AIETP C3; insertion of the extra thiophene moiety is intended to provide an extended π conjugation which can be instrumental in red-shifting the absorption and emission maxima of the probes (Scheme 1). On the other hand, to study the effect of structural variation on aqueous-dispersible nanoparticle formation ability, a phenyl-thiazole unit has been incorporated in AIETP, AIETP C2 and AIETP C3 instead of lone phenyl moiety in AIETP C1. Moreover, control compound AIETP C3 has been compared with the rationally designed two-photon probe AIETP to reflect the effect of -Br substitution on the photostability. All the compounds have been well characterized with the NMR and mass spectra (Figure S1-17, Supporting Information). Subsequently, the photo-physical properties of the probes were studied in details.

**Figure 1:**
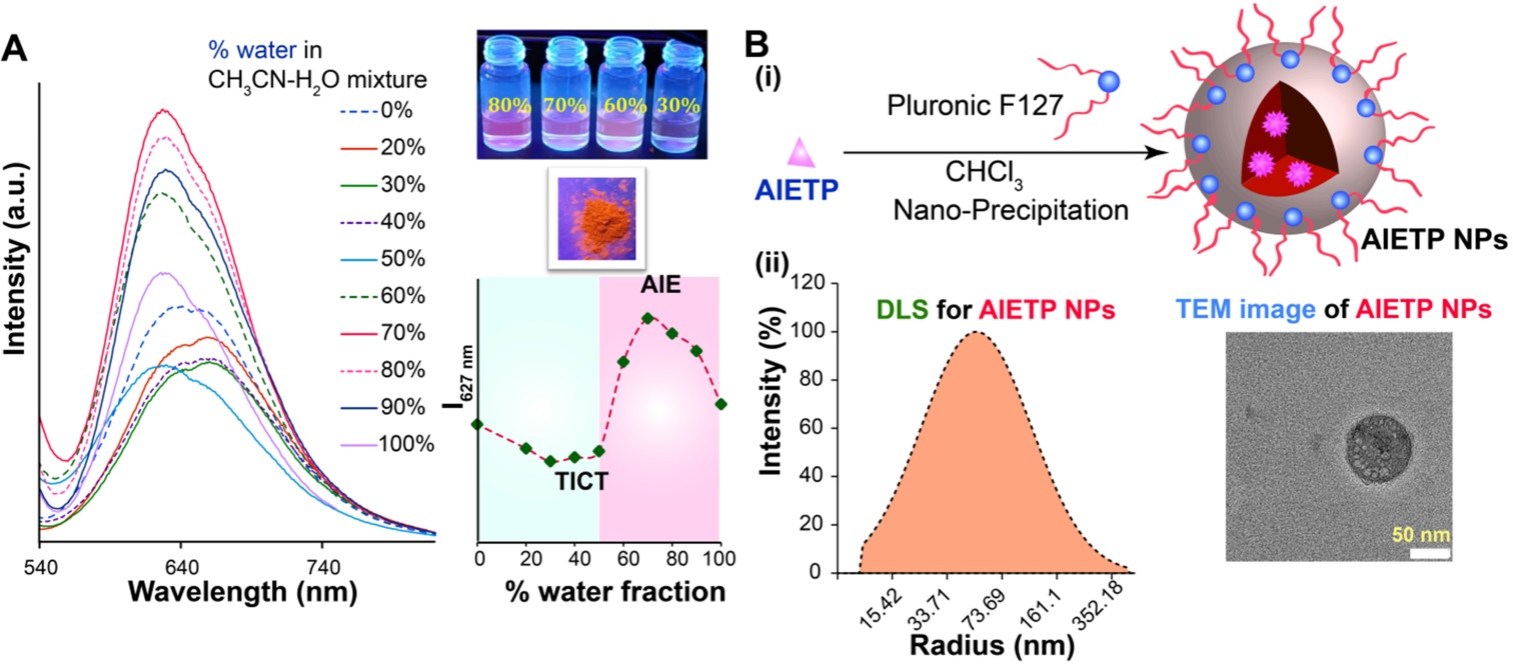
(A) Emission spectra of AIETP (10 µM) upon changing the water fraction of CH_3_CN-water mixed solvent; λ_ex_ = 520 nm. Inset: visual changes in fluorescence (under UV light) with different water fractions, solid state emission under UV light and the changes in the emission intensity at 627 nm with different water fractions; (B) (i) Schematic representation of nanoparticle formation procedure; (ii) Characterization of the AIETP NPs *via* DLS and TEM analysis (scale bar 50 nm).

The UV-Vis spectra of the probe AIETP was recorded in aqueous PBS buffer medium (containing 0.2% DMSO) which revealed a broad absorbance maximum in the region of 480-520 nm (Figure S18, Supporting Information). Extensive fluorescence spectral studies of the fluorescent probe AIETP in CH_3_CN-water medium was carried out to confirm the AIE activity. AIETP revealed a clear emission peak at 620 nm with reasonable intensity when excited at 520 nm, which can be attributed to the presence of TICT (Figure 1A). Upon increasing the water fraction up to 50% there was gradual decrease in the emission intensity of the probe owing to the predominance of TICT process. Further enhancement of the water fraction witnessed dramatic increase in the emission intensity with an emission peak at 627 nm (Figure 1A), indicating the occurrence of aggregation induced emission (AIE). However, the probe in more than 90% water fraction (up to ~100%) experiences slight decrease in the emission intensity which can be attributed to the precipitation of the probe and decrease in the local concentration. Therefore, the probe can clearly demonstrate the characteristic AIE features in CH_3_CN-water medium wherein the TICT phenomenon is also pertinent at lower water fraction. The presence of both the TICT and AIE features in the single probe could provide the proper combination to explore it in efficient two-photon imaging studies as envisioned previously.

The probe is also strongly emissive in the solid state as captured under the UV-light illumination (Figure 1A, inset), which also validated the AIE feature of the probe. Meanwhile, the control compounds AIETP C1, AIETP C2 and AIETP C3 also demonstrated similar trend in the fluorogenic behavior and exhibited AIE properties in CH_3_CN-water medium as depicted in the Figure S19-21 (Supporting Information). However, the solubility of the AIE-gens in aqueous medium is not suitable for their potential application in the bioimaging. Therefore, we tried to synthesize aqueous dispersible nanoparticles of these probes by encapsulating into polymer Pluronic F127.

### Fabrication and characterization of the AIE nanoparticles

A modified well-known nanoprecipitation method was followed to synthesize the nanoparticles. The schematic representation of the nanoparticle formation procedure for the AIE probes is clearly depicted in Figure 1B(i). The detailed procedure of the AIE nanoparticles fabrication (Scheme S2, Supporting Information) has been also included in the Experimental Section. In brief, AIE probes and Pluronic F127 were dissolved in CHCl_3_ and sonicated well to prepare the homogeneous solutions. The CHCl_3_ was then evaporated carefully from all these homogeneous solutions to obtain precipitated residues. Water was subsequently added to each sample followed by the prolonged sonication to dissolve/disperse these residues. Interestingly, the precipitated residues for AIETP, AIETP C2 and AIETP C3 dissolved/dispersed frequently in water whereas that of the AIETP C1 mostly remained undissolved. The resultant aqueous solutions for all the AIE probes were filtered through 0.45 µm filters. Filtration resulted in the clear deep red, orange and red solutions respectively for AIETP, AIETP C2 and AIETP C3 indicating towards the likely formation of corresponding aqueous-dispersible AIE-nanoparticles (NPs). However, in case of AIETP C1 the filtration failed to acquire such solution which might be due to the very poor solubility/dispersibility of the resultant (AIETP C1-Pluronic F127) mixture in water, validating the lack of aqueous dispersibile nanoparticle formation ability. Therefore, it is clearly evident from these results that the structural variations can substantially influence the nanoparticle fabrication aptitude of AIE probes with amphiphilic polymers such as Pluronic F127 (Scheme 1). In the present study, the incorporation of the phenyl-thiazole unit in AIETP, AIETP C2 and AIETP C3 certainly facilitated water-dispersible nanoparticle formation over the presence of only phenyl moiety in AIETP C1. Incorporation of the phenyl-thiazole moiety possibly maintained a proper hydrophobic/hydrophilic balance with the Pluronic F127 that enabled the nanoprecipitation method to fabricate the water-dispersible nanoparticles (AIETP NPs, AIETP C2 NPs and AIETP C3 NPs) while the presence of phenyl moiety instead could not meet the suitable condition (AIETP C1 NPs*: very poor dispersibility in water). A UV-Vis spectral study also indicated that the synthesized nanoparticles AIETP NPs, AIETP C2 NPs and AIETP C3 NPs have far better dispersibility in water than their parent compounds as the relative baselines of the UV-Vis spectra of AIETP, AIETP C2 and AIETP C3 were found to be much higher than their nanoparticle counterparts (Figure S22, Supporting Information). On the contrary, in case of AIETP C1 NPs*, there was huge rise in baseline of the UV-Vis spectra compared to that of the AIETP C1, which evidently reiterated again the very poor dispersibility of AIETP C1 NPs* in aqueous medium (Figure S22, Supporting Information).

AIETP NPs were characterized by dynamic light scattering (DLS), transmission electron microscopy (TEM) and scanning electron microscope (SEM) studies. AIETP-NPs rendered average particle size of 45 nm radius (Figure 1B(ii), left). The TEM and SEM images of AIETP-NPs also confirmed the round shaped uniform nanoparticle formation (Figure 1B(ii), right and Figure S23, Supporting Information). Hence, the probe AIETP has the ability to form highly water-dispersible well characterized NPs with Pluronic F127. It might be highlighted here that AIETP C1 NPs* could not exhibit similar nanoparticle fabrication and the resultant aqueous suspension lead to ununiform larger particle size which was corroboroted by the corresponding SEM and TEM images (Figure S23-24, Supporting Information). Formation of AIETP C2 NPs and AIETP C3 NPs were also confirmed by the SEM and TEM analysis and the corresponding TEM and SEM images of the AIETP C2 NPs and AIETP C3 NPs have been presented in the supporting information (Figure S23-24, Supporting Information). Therefore, the aqueous solution of the nanocomposites of the AIETP NPs, AIETP C2 NPs and AIETP C3 NPs were subjected to the two-photon imaging studies. It is also worth mentioning that the dye (AIETP) encapsulation efficiency (EE) and the concentration of the AIETP NPs were calculated (details in the Supporting Information) to be 99% (Figure S25, Supporting Information) and 4.35 nM respectively.

### Two-photon properties and the photostability of AIE nanoparticles

AIETP NPs, AIETP C1 NPs*, AIETP C2 NPs and AIETP C3 NPs were excited using the two-photon femtosecond laser at different wavelengths and the two-photon emission intensities were recorded accordingly (Figure S26, Supporting Information). It was interesting to note that in case of AIETP NPs, the very high emission intensity could be observed when excited at 1040 nm (Figure S26, Supporting Information). AIETP C1 analogue has very poor dispersibility in water and hence depicted very less two-photon emission intensities where the best attainable emission intensity was obtained around 860 nm two-photon excitation. The AIETP C2 NPs and AIETP C3 NPs also revealed moderate emission intensities with two-photon laser excitation at 950 nm and 1040 nm respectively. Therefore, the aqueous soluble nanoparticles of the two-photon probes AIETP and AIETP C3 can only afford the excitation at NIR-II region which is advantageous for *in vivo* imaging applications. Other two-photon probes (AIETP C1 and AIETP C2) could only utilize excitation in the NIR-I (700-1000) range (Figure S26, Supporting Information) and these probes were not studied further.

Among the NIR-II excitable two-photon probes, AIETP NPs exhibited much brighter emission than AIETP C3 NPs under same experimental conditions when excited at 1040 nm using two-photon femtosecond laser (Figure S26, Supporting Information). It might be mentioned here that structurally the probes AIETP and AIETP C3 are only differed by the presence of an extra -Br substitution in the phenyl-thiazole unit of AIETP whereas AIETP C3 lack that -Br unit (Scheme 1). It was interesting to note that upon continuous two-photon laser irradiation (1040 nm) AIETP NPs rendered much better photostability than AIETP C3 NPs (Figure S27, Supporting Information). Therefore, the extra photo-stability or better anti-photobleaching capability of AIETP under 1040 nm two-photon laser irradiation could be attributed to the insertion of -Br substitution in the structural feature (Scheme 1) which is indispensable for efficient long-term bioimaging studies. Probably, compared to the heavy-atom effect induced by the introduction of 'Br' atom that enables the spin-orbit coupling to promote the intersystem crossing between singlet and triplet states, the inter molecular halogen bonding (Br···Br interaction) might have played more significant role to facilitate the partial restriction of nonradiative decay to ensure better photostability of AIETP 34. Additionaly, AIETP NPs also revealed better resistance to the photobleaching compared to the commercial fluorescent dyes such as Evans blue and Alexa 647 upon 1040 nm continuous two-photon laser irradiation (Figure S27, Supporting Information), which again reiterated the high photo-stability of AIETP NPs. It is pertinent to mention here that the AIETP NPs possessed higher relative fluorescence quantum yield (0.060) than the native AIETP (0.039) and rendered much brighter fluorescence (Figure S28, Supporting Information).

**Figure 2:**
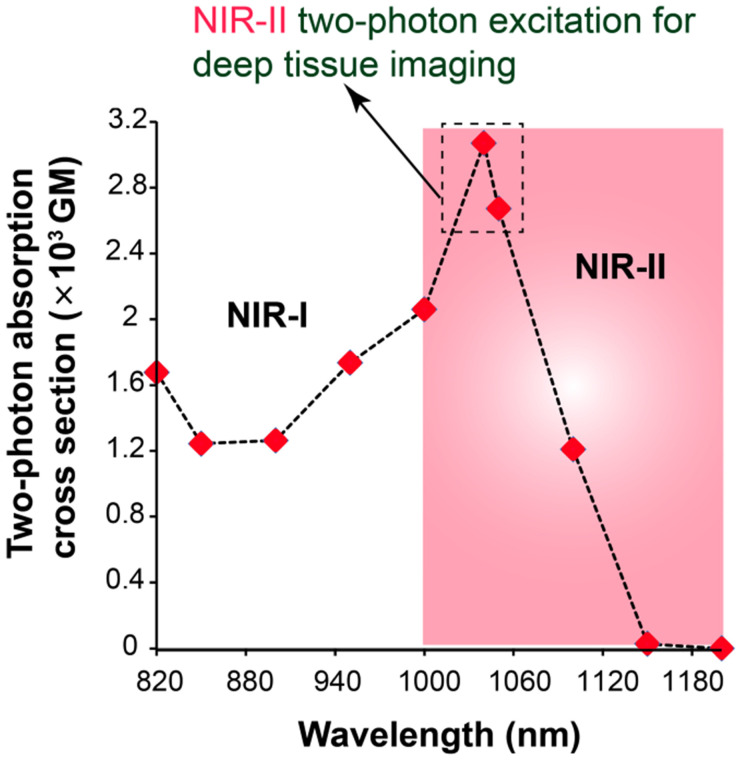
Two-photon absorption cross section of AIETP NPs at different two-photon excitations.

Two-photon absorption cross section (σ_2_) is a very important feature of a fluorescent probe that determines the two-photon excitation efficacy of the particular fluorophore for two-photon imaging. The σ_2_ of AIETP NPs in aqueous media was calculated within the excitation wavelength range from 820 to 1200 nm using a two-photon induced fluorescence method at 20 nm intervals (Figure 2). Rhodamine B was selected as the standard for the calculation of the σ_2_ values of AIETP. The two-photon cross section plot in different wavelengths revealed that AIETP NPs have excellently high TP cross section at around 1040-1050 nm (Figure 2). Two-photon absorption cross section (σ_2_) of AIETP C2 NPs and AIETP C3 NPs were also calculated using same method which reveled that AIETP NPs possess much higher σ_2_ than AIETP C2 NPs or AIETP C3 NPs at 1040 nm (Figure S29, Supporting Information). Therefore, the AIETP NPs is most suitable for the NIR-II excitable two-photon imaging studies and hence, the *in vivo* imaging aptitude of the AIETP NPs has been studied extensively.

### Biocompatibility and phototoxicity of AIETP NPs

The cytotoxicity is an important factor that decides the applicability of a fluorescent probe in live cell/species imaging studies. In this regard, AIETP NPs showed very good biocompatibility as established by the cytotoxicity study. It revealed that incubation of the probe even at a very high concentration (150 µM) in live HeLa cells for 24 h could exert very minimal cytotoxicity (Figure S30, Supporting Information). Essentially, upon two-photon excitation, AIETP NPs displayed very bright two-photon fluorescence inside live cells (Figure S31, Supporting Information). Apart from having a very good aqueous dispersibility; AIETP NPs also rendered good cell permeability. It should also be highlighted here that AIETP NPs exhibited negligible *in vivo* toxicity to live mice under the experimental condition. Administration of AIETP NPs did not inflict any noticeable abnormality in the major organs (brain, kidney, lung, spleen, liver, and heart) of live mice as confirmed by the histological examination (Figure S32, Supporting Information). Hence, the photostability and biocompatibility of the NIR-II excitable two-photon probe AIETP NPs are appropriate for exploring it in the *in vivo* bioimaging studies with high depth penetration such as brain imaging. However, it is very important to find out whether the AIETP NPs form reactive oxygen species (ROS) in presence of two-photon excitation light or not; which can impede the long-term bioimaging by introducing notorious photo-toxicity. It is worth mentioning that AIETP NPs depicted negligible ROS generation capabilities under 1040 nm irradiation even though typically many AIE probes are vulnerable to ROS generation 35, 36. As shown in Figure S33 (Supporting Information), the cells treated with AIETP NPs and ROS marker 2′,7′-dichlorofluorescin diacetate (DCFDA) could not reveal any fluorescence in green channel even after 120 scans with two-photon excitation (DCFDA labeled cells show green fluorescence in presence of ROS; Figure S34, Supporting Information). This result indeed indicated the minimal ROS generation capability of AIETP NPs upon two-photon excitation light irradiation and endorsed the feasibility of safely utilizing AIETP NPs for live *in vivo* model. It might be highlighted that unlike the two-photon AIE systems wherein certain probe-protein hybrids are fabricated to minimize the phototoxicity; AIETP NPs are self-sufficient to exhibit negligible phototoxicity 37.

### NIR-II excitable intravital two-photon imaging of brain vasculature with AIETP NPs

AIETP NPs with high biocompatibility, good chemical stability and photo-stability revealed bright far-red/NIR fluorescence emission under NIR-II two-photon excitation at 1040 nm which is ideal for utilizing it as a promising fluorogenic system for robust two-photon *in vivo* imaging. To elucidate the deep imaging aptitude of the probe, a craniotomy-based mouse brain model was applied. Basically, the head hair of the mouse was removed and skin incision was performed with outmost care to expose the skull followed by the removal of a 3 mm-diameter skull bone via microsurgery (a dental drill) to perform the *in vivo* two-photon brain imaging experiment (Scheme S3-5, Supporting Information). The details of the experimental procedure can be found in experimental section (Scheme S3-5, Supporting Information). Subsequently, 200 µL of the prepared aqueous solution of the AIETP NPs (4.35×10^-9^ M) was injected into the mouse using retro-orbital injection. On a stereotactic setup that comprises body temperature maintaining heating pad, the head of the mouse was properly positioned and immobilized for two-photon microscopy. Two-photon excitation at ~1040 nm wavelength was used for two-photon *in vivo* imaging studies since the largest σ_2_ value of AIETP NPs was achieved at 1040 nm (Figure 2).

**Figure 3:**
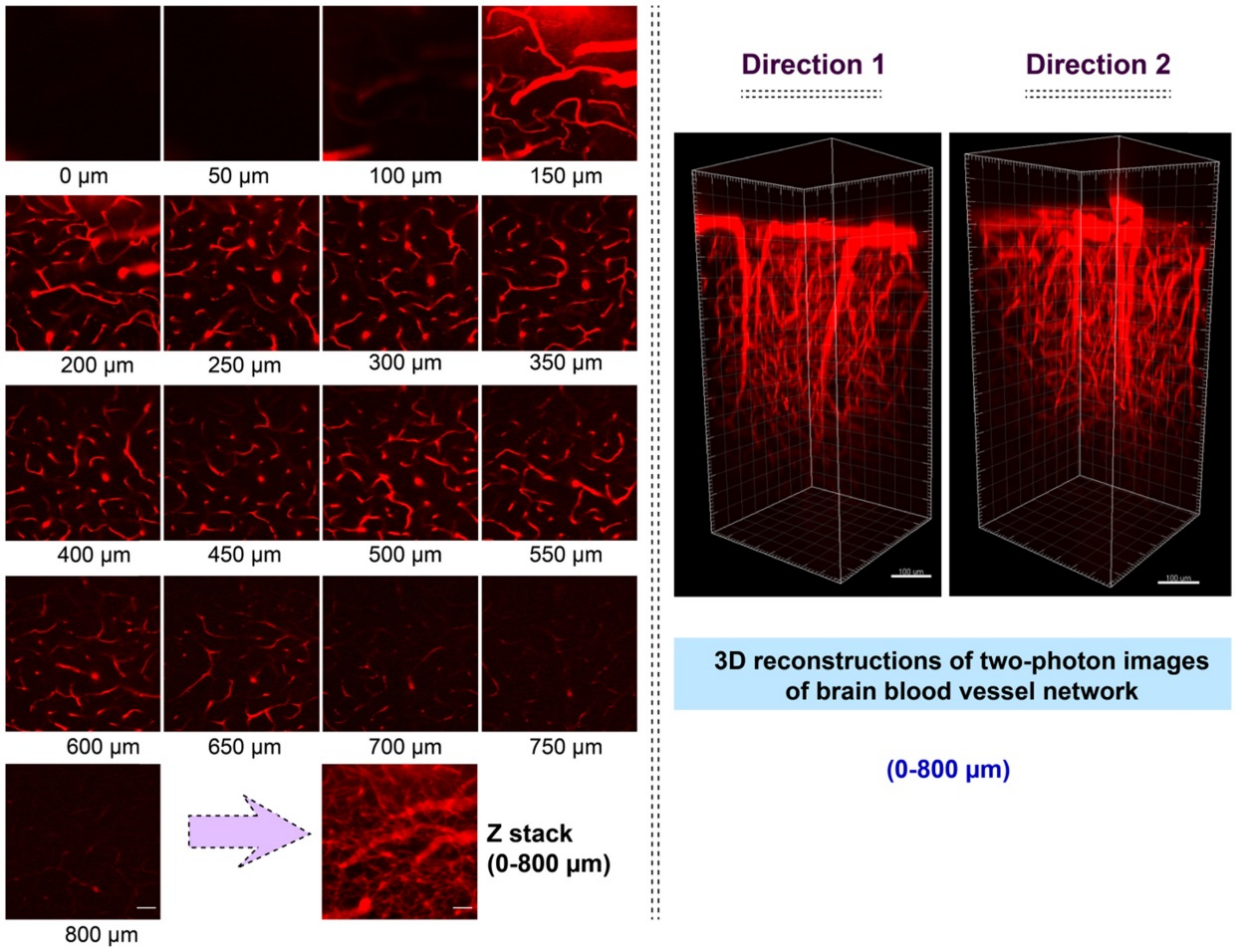
Intravital two-photon fluorescence imaging of mouse brain vasculature network labelled with AIETP NPs. (Left) Two-photon fluorescence images of the brain vasculatures at various depths under NIR-II (1040 nm) excitation, Scale bar for all images: 50 µm; (Right) Reconstructed 3D two-photon images of the mouse brain blood vessel network at 0 to 800 µm depth under NIR-II (1040 nm) excitation from different directions. Scale bar: 100 µm.

The bright two-photon images of brain vasculatures labelled with AIETP NPs were captured at different tissue depths (Figure 3). Even though the AIETP NPs can label the whole brain vasculature network (Scheme S4, Supporting Information), the specific brain region, which was selected to extensively monitor the brain vasculature images (in Figure 3) has been indicated in the Scheme S3 (Supporting Information). AIETP NPs were found to be stable enough throughout the circulation in the blood vessels and rendered bright emission. The clear high-resolution brain vasculature network at various depth penetrations can be clearly visualized from the two-photon images (Figure 3, Left). The two-photon images of the same brain area at different depths clearly revealed the brain blood vessels comprising both the tiny capillaries in the deep brain layer as well as the big blood vessels in the superficial brain layer.

Therefore, the brain imaging with AIETP NPs can certainly afford the flexibility in distinguishing big blood vessels from the small blood capillary vessels at varying depths. It might be highlighted here that a large maximal brain imaging depth of 800 μm was achieved (Figure 3, Left). This deeper imaging depth, compared to the several other two-photon probes can be attributed to reduced light attenuation by the highly scattering brain tissue upon NIR-II light excitation apart from considering the good two-photon cross section and high brightness of the AIETP NPs in aqueous medium 38, 39.

NIR-II light excitability of AIETP NPs in aqueous medium also became instrumental in acquiring the higher contrast *in vivo* two-photon imaging with high resolution owing to the low background noise. The signal-to-background ratios (SBR) for the obtained *in vivo* two-photon images of the brain vasculature network at different imaging depth were calculated to realize the imaging contrast (Figure S35, Supporting Information). High SBR values (>5) (Figure S35, Supporting Information) of the two-photon images at different imaging depth clearly validated the high-contrast* in vivo* deep brain vasculature imaging capability of AIETP NPs. Also, the line intensity profile across a tiny blood vessel at penetration depth of 800 μm was plotted to evaluate the spatial resolution, which revealed the full width at half maximum (FWHM) to be around 1.92 μm (Figure S36, Supporting Information). This very high spatial resolution made AIETP NPs an outstanding choice for brain imaging wherein the obtained resolution is much better compared to several other recently reported probes 5, 40-43. Some recently reported multi-photon fluorescent probes have been summarized in Table S1 (Supporting Information) to compare their imaging efficacy with that of the newly developed fluorescent probe AIETP NPs in terms of penetration depth and spatial resolution. It is also important to monitor whether AIETP NPs could enter the brain tissue by crossing the brain blood vessels wall or not. To validate that the two-photon images of the mouse brain blood vessels were captured 48h after the administration of AIETP NPs. It is worth mentioning that even after 48h of the administration of AIETP NPs no obvious fluorescence signal could be observed outside the blood vessel which reiterated good stability of AIETP NPs in blood stream (Figure S37, Supporting Information). This result could be attributed to the blood-brain barrier (BBB) which prevents the exogenous agents from entering into the brain to safeguard its regular functions.

The two-photon NIR-II excitable bright fluorescence of AIETP NPs with good stability also made it possible to visualize the 3D image of brain vasculature, owing to the inherent 3D section property of two-photon excitation. At several tissue depths the 3D two-photon fluorescence images of the brain vasculature structure network have been reconstructed from the obtained NIR-II excitable two-photon images (Figure 3, Right). The reconstructed 3D two-photon fluorescence images could evidently reveal the brain vasculature structure network up to 800 μm depth by capturing the distribution of AIETP NPs in real time. The 360-degree movie of the reconstructed images at different depths has been incorporated as the supporting video file (for web version only) for the convenience. These outcomes validate that NIR-II excitable AIETP NPs can eloquently serve as a promising two-photon probe for high contrast brain vasculature imaging with remarkable penetration depth.

## Conclusion

In summary, this work highlights the judicious fluorescent probe design strategy to develop efficient NIR-II excitable AIE active two-photon probe with improved photostability, high brightness and good water-soluble nanoparticle fabrication ability for robust deep penetration *in vivo* imaging. The effect of structural modulations in the nanoparticle fabrication ability of the probe with polymer has been rationally studied which clearly outlined the scope of designing newer aqueous soluble nanocomposite systems. Among the developed probes, the rationally designed probe AIETP was capable of showing better photostability and effectively formed aqueous soluble nanoparticle AIETP NPs with Pluronic F127. AIETP NPs revealed very good two-photon cross section and *in vivo* stability apart from demonstrating very good cell permeability and biocompatibility which facilitated its use in the *in vivo* two-photon imaging. NIR-II excitable AIETP NPs were utilized to capture the 2D and 3D high contrast two-photon brain vasculature imaging with outstanding penetration depth of 800 μm and a very high spatial resolution of 1.92 μm. This work outlines the great promise in designing newer two-photon AIE probes with good aqueous soluble nanoparticle formation aptitude for efficient high resolution, high contrast deep *in vivo* brain imaging under NIR-II light excitation in future.

## Supplementary Material

Supplementary figures and tables.
