# Supplementary material for: AIE-active two-photon fluorescent nanoprobe with NIR-II light excitability for highly efficient deep brain vasculature imaging
Source: Theranostics. 2021 Jan 1;11(5):2137–48. doi: 10.7150/thno.53780 (PMC7797691; doi:10.7150/thno.53780)
Supplement: Supplementary file 1 — Supplementary figures and tables. [file thnov11p2137s1.pdf]

Supporting Information

AIE-Active two-photon fluorescent nanoprobe with NIR-II light excitability for highly efficient deep brain vasculature imaging

Soham Samanta,[§] Meina Huang,[§] Shaoqiang Li,[§] Zhigang Yang*, Ying He, Zhenyu Gu, Jianguo Zhang, Dan Zhang, Liwei Liu and Junle Qu*

Center for Biomedical Photonics & Key Laboratory of Optoelectronic Devices and Systems of Guangdong Province and Ministry of Education, College of Physics and Optoelectronic Engineering, Shenzhen University, Shenzhen 518060, China
E-mail: zhgyang@szu.edu.cn, jlqu@szu.edu.cn
[§] *Equal contributions*

Table of Contents Pages

Experimental Procedures [General Information and Materials, Synthesis and characterization of the fluorescent probes, Fabrication of nanoparticles, Determination dye encapsulation efficiency of AIETP NPs, Determination of Concentration of AIETP NPs, Fluorescence quantum yield measurement, Two-photon properties and the two-photon cross section, Cytotoxicity and cell imaging studies of AIETP NPs, Intracellular ROS Evaluation, In vivo brain imaging studies, Selecting the area for brain imaging, *In vivo* toxicity] ………………………………………………………………………… **S1-S6**

Scheme S1 Synthesis of the two-photon AIE probes…………………………………………… **S2**
Scheme S2 Schematic illustration of the fabrication of nanoparticles ……………………… **S3**
Scheme S3 Specific brain region for in vivo imaging………………………………………… **S5**
Scheme S4 Whole brain labelling aptitude of AIETP NPs …………………………………… **S5**
Scheme S5 Reason for choosing specific portion for brain imaging ………………………… **S6**
Figure S1 ^{1}H-NMR spectra of AIETP in DMSO-d_6…………………………………………… **S7**
Figure S2 ^{13}C-NMR spectra of AIETP in $CDCl_3$ …………………………………………… **S7**
Figure S3 Expanded ^{13}C-NMR spectra of AIETP in $CDCl_3$ ………………………………… **S8**
Figure S4 Mass spectrum of AIETP…………………………………………………………… **S8**
Figure S5 ^{1}H-NMR spectra of AIETP C1 in $CDCl_3$……………………………………………… **S9**
Figure S6 Expanded (aromatic region) ^{1}H-NMR spectra of AIETP C1 in $CDCl_3$……………… **S9**
Figure S7 Mass spectrum of AIETP C1 ……………………………………………………… **S10**
Figure S8 ^{1}H-NMR spectra of AIETP C2 in $CDCl_3$ ……………………………………………**S10**
Figure S9 Expanded (aromatic region) ^{1}H-NMR spectra of AIETP C2 in $CDCl_3$……………… **S11**
Figure S10 ^{13}C-NMR spectra of AIETP C2 in $CDCl_3$ ………………………………………… **S11**
Figure S11 Expanded ^{13}C-NMR spectra of AIETP C2 in $CDCl_3$ ……………………………… **S12**
Figure S12 Mass spectrum of AIETP C2 …………………………………………………… **S12**
Figure S13 ^{1}H-NMR spectra of AIETP C3 in $CDCl_3$ ……………………………………………**S13**
Figure S14 Expanded (aromatic region) ^{1}H-NMR spectra of AIETP C3 in $CDCl_3$ ……………**S13**
Figure S15 ^{13}C-NMR spectra of AIETP C3 in $CDCl_3$ ………………………………………… **S14**
Figure S16 Expanded ^{13}C-NMR spectra of AIETP C3 in $CDCl_3$ ……………………………… **S14**
Figure S17 Mass spectrum of AIETP C3 ……………………………………………………**S15**
Figure S18 UV-Visible spectra of AIETP in aqueous PBS ……………………………………**S15**
Figure S19-21 AIE properties of AIETP C1, AIETP C2 and AIETP C3……………………………**S16-17**
Figure S22 Changes in UV-Visible spectra upon nanoparticle fabrication……………………**S17**
Figure S23-24 SEM and TEM images of NPs…………………………………………………… **S18**
Figure S25 Absorption calibration curve of AIETP……………………………………………… **S19**
Figure S26 Two-photon fluorescence intensities ……………………………………………… **S19**
Figure S27 Photostability of AIETP NPs, AIETP C3 NPs, Evans blue and Alexa 647 …………**S20**
Figure S28 Change in fluorescence spectra of AIETP upon nanoparticle fabrication…………**S20**
Figure S29 Two-photon absorption cross section of AIETP NPs, AIETP C2 NPs and AIETP C3 NPs……**S21**
Figure S30 CCK-8 cytotoxic assay of AIETP NPs ……………………………………………**S21**
Figure S31 Two-photon fluorescence imaging of live HeLa cells with AIETP NPs ……………**S21**
Figure S32 Histological examination of mice major organs……………………………………**S22**
Figure S33 Evaluation of AIETP NPs induced intracellular ROS generation …………………**S22**
Figure S34 ROS control study …………………………………………………………………**S23**
Figure S35 Signal-to background ratio (SBR) of the two-photon brain vasculature images……**S23**
Figure S36 FWHM Plot to determine the resolution of the two-photon image at 800 μm depth ………**S24**
Figure S37 In vivo stability of AIETP NPs in brain blood stream ……………………………**S24**
Table S1 Comparing the two-photon fluorescent probes for brain vasculature imaging……………**S25**

Experimental Procedures

General Information and Materials: For the synthesis of fluorescent probes, all the starting materials were purchased from commercial suppliers and used without further purification. Other chemicals for biological experiments and analytical studies (such as phosphate buffered saline (1×, pH: 7.4), 2′,7′-dichlorofluorescin diacetate (DCFDA), Rhodamine B etc.) were also bought from authentic commercial suppliers. To record the absorption spectra, a Cintra 2020 UV−Vis spectrophotometer was used and the spectral data were recorded in the range of 300−800 nm wavelength using quartz cuvettes with 10 mm path length at 298 K. Similarly, the fluorescence measurements were performed on a Horiba Fluoromax-4 spectrofluorometer using 10 mm path length quartz cuvettes with a slit width of 3 nm at 298 K. Dynamic light scattering (DLS) studies were conducted in a Brookhaven Instrument to analyse the particle size. Transmission electron microscopy (TEM) analysis was performed with a F200 TEM instrument. The mass spectra of the fluorescent probes were obtained using ThermoScientific Q Exactive mass spectrometer and the mass samples of the probes were prepared in acetonitrile. A drop of the diluted trifluoroacetic acid (TFA) solution was added to each sample for ionization before acquiring the mass spectra since the probes are uncharged. The Nuclear magnetic resonance (NMR) spectra were recorded on a 400 MHz BRUKER AVANCE 300 instrument. The chemical shifts were recorded in parts per million (ppm) on the scale. The spin multiplicities in ^{1}H NMR spectra are described using the following abbreviations: s = singlet; d = doublet; t = triplet; q = quartet; m = multiplet.

Synthesis and characterization of the fluorescent probes:

AIETP: At first, 5-(4-(diphenylamino)phenyl)thiophene-2-carbaldehyde was synthesized by the Suzuki coupling reaction. 11 mmol (1.71 g) of (5-formylthiophen-2-yl) boronic acid and the 10 mmol (3.24 g) of 4-Bromotriphenylamine compound were dissolved in 35 mL of dioxane in a round bottom flask. Subsequently, 0.55 mmol of $Pd(PPh_3)_4$ and 20 mmol of Na_2CO_3 (aqueous solution) were added to it under a nitrogen atmosphere and the reaction mixture was refluxed for 24h with continuous stirring. A dark black reaction mixture was obtained which was filtered after it cooled down to room temperature. To the filtrate, DCM (50 mL) and water (30 mL) were added and the organic layer was separated using separating funnel. It was dried over the $MgSO_4$ and the solvent was then evaporated to get the crude product. The final pure yellow solid product of 5-(4-(diphenylamino)phenyl)thiophene-2-carbaldehyde was then obtained from the column chromatography (eluent EtOAc/Hexane in the ratio of 5:95) (Scheme S1, 1st step).

In the next step, 2.0 mmol of 5-(4-(diphenylamino)phenyl)thiophene-2-carbaldehyde and 2.0 mmol of 4-(4-Bromophenyl)-2-thiazoleacetonitrile were dissolved in EtOH. The resultant mixture was then refluxed in presence of 3.0 mmol NaOAc for 12 h to yield the brown-red product of AIETP (Scheme S1). The product was filtered, washed with methanol several times and dried in open air.

Calculated yield (for the final step): 86%. ^{1}H NMR [400 MHz, DMSO-d_6, TMS, J (Hz), δ (ppm)]: 8.52 (1H, s), 8.30 (1H, s), 7.99 (2H, d, J = 6.8), 7.94 (1H, d, J = 3.2), 7.69-7.67 (4H, m), 7.62 (1H, d, J = 3.2), 7.37 (4H, t, J = 6.4), 7.14 (2H, d, J = 6.0), 7.11 (4H, d, J = 6.4), 6.99 (2H, d, J = 6.8). ^{13}C NMR [100 MHz, $CDCl_3$, TMS, δ (ppm)]:162.9, 155.7, 152.2, 149.0, 147.1, 137.0, 136.8, 135.1, 132.9, 132.1, 129.6, 128.2, 127.7, 127.3, 126.4, 125.2, 123.9, 123.2, 122.7, 122.6, 113.9, 99.8. ESI-MS (positive mode, m/z) Calculated for $C_{34}H_{23}BrN_3S_2$: 616.0512, 618.0491. Found: 618.0487 [(M + H^+)].

AIETP C1: First, 5-(4-(diphenylamino)phenyl)thiophene-2-carbaldehyde was synthesized following the same procedure mentioned in the detailed synthetic procedure of AIETP . In the final step, 1.0 mmol of 5-(4-(diphenylamino)phenyl)thiophene-2-carbaldehyde and 1.0 mmol of 2-(4-bromophenyl)acetonitrile were refluxed in EtOH in presence of 1.5 mmol NaOAc for 10 h to yield the orange solid product of the control compound AIETP C1 which was then filtered, washed several times with cold methanol and dried in open air (Scheme S1).

Calculated yield (for the final step):79%. ^{1}H NMR [400 MHz, $CDCl_3$, J (Hz), δ (ppm)]: 7.59 (1H, s), 7.57-7.49 (6H, m), 7.57-7.49 (7H, m), 7.57-7.49 (7H, m). ESI-MS (positive mode, m/z) Calculated for $C_{31}H_{22}BrN_2S$: 533.0682, 535.0662. Found: 533.0682, 535.0658 [(M + H^+)].

AIETP C2: In a round bottom flask, 1.0 mmol of 4-(diphenylamino)benzaldehyde and 1.0 mmol of 4-(4-Bromophenyl)-2-thiazoleacetonitrile were dissolved in dry EtOH. The resultant mixture was refluxed in presence of 1.5 mmol NaOAc for 17 h to yield the orange-yellow solid product of AIETP C2 (Scheme S1). The reaction mixture was allowed to come to the room temperature and the precipitate was filtered. The orange-yellow solid product was washed with cold methanol several times and dried in open air.
Calculated yield (for the final step): 88%. ^{1}H NMR [400 MHz, $CDCl_3$, *J* (Hz), δ (ppm)]: 8.10 (1H, s), 7.86 (4H, t, *J* = 8.0), 7.57 (2H, d, *J* = 8.4), 7.49 (1H, s), 7.36 (4H, dd, *J* = 8.4), 7.19-7.15 (6H, m), 7.04 (2H, d, *J* = 8.8). ^{13}C NMR [100 MHz, $CDCl_3$, TMS, δ (ppm)]:163.9, 156.5, 151.3, 146.2, 144.1, 133.0, 132.0, 131.9, 129.8, 129.7, 128.2, 126.3, 125.1, 124.8, 122.6, 120.1, 117.8, 113.5. ESI-MS (positive mode, m/z) Calculated for $C_{30}H_{21}BrN_3S$: 534.0635, 536.0614. Found: 534.0634 [(M + H^+)].

AIETP C3: 1.0 mmol of 5-(4-(diphenylamino)phenyl)thiophene-2-carbaldehyde (synthesized following first step of Scheme S1) and 1.0 mmol of 4-phenyl-2-thiazoleacetonitrile were dissolved in dry EtOH and the resultant mixture was refluxed with continuous stirring in presence of NaOAc for 20 h to yield the orange-red product of the control compound AIETP C3. The solid product was filtered after the reaction mixture came to the room temperature (Scheme S1). It was then washed thoroughly with methanol and dried in open air.
Calculated yield (for the final step): 53%. ^{1}H NMR [400 MHz, $CDCl_3$, J (Hz), δ (ppm)]: 8.34 (1H, s), 7.97 (2H, d, *J* = 7.2), 7.68 (1H, d, *J* = 4.4), 7.55-7.50 (3H, m), 7.47 (2H, t, *J* = 7.2), 7.39 (1H, t, *J* = 7.2), 7.32-7.28 (5H, m), 7.15-7.05 (8H, m). ^{13}C NMR [100 MHz, $CDCl_3$, TMS, δ (ppm)]:162.6, 156.9, 151.9, 148.9, 147.1, 136.9, 136.6, 135.2, 134.0, 129.6, 128.9, 128.7, 127.2, 126.6, 126.4, 125.2, 123.9, 123.1, 122.6, 117.5, 113.6, 99.9. ESI-MS (positive mode, m/z) Calculated for $C_{34}H_{24}N_3S$: 538.1407. Found: 538.1404 [(M + H^+)].

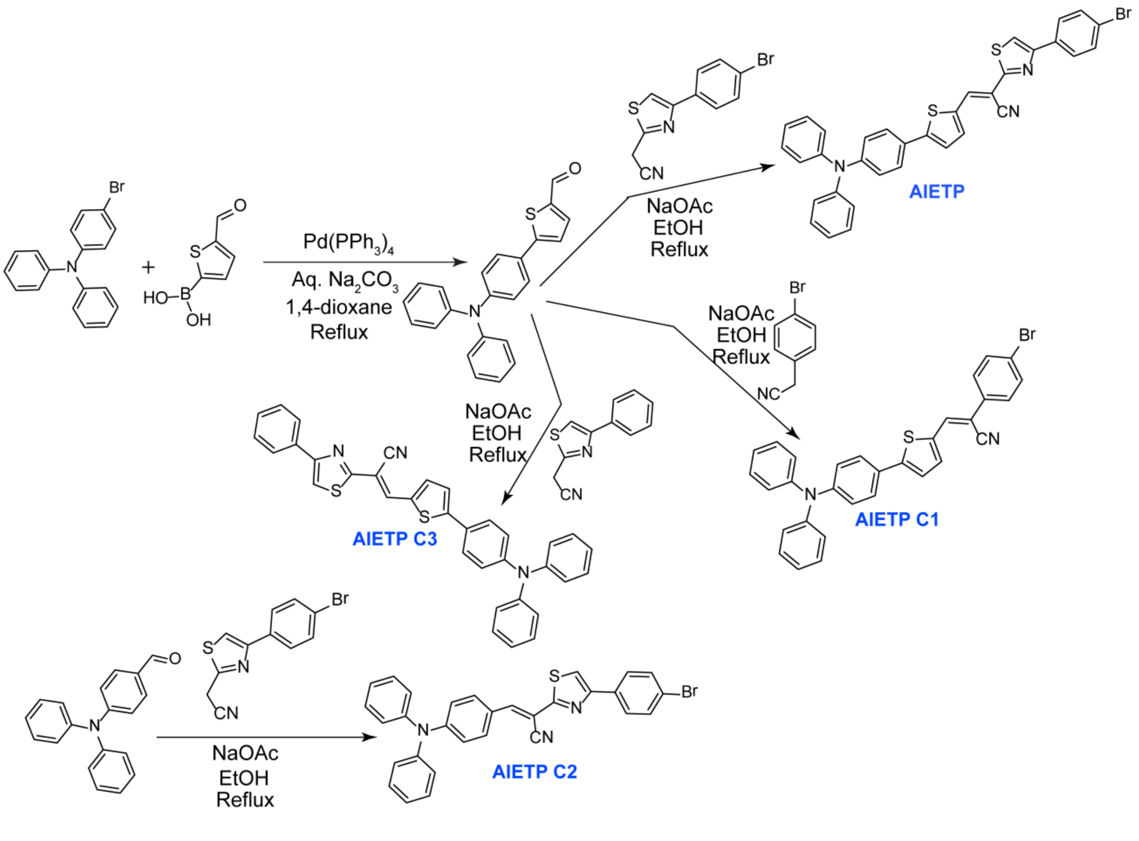

Scheme S1. Synthesis of the two-photon AIE probes.

Fabrication of nanoparticles: The well-documented nanoprecipitation technique with slight modifications was followed to synthesize the nanoparticles for the AIE probes. Briefly, in each of the four different vials 1 mg of AIETP or AIETP C1 or AIETP C2 or AIETP C3 was dissolved

in 1 mL of $CHCl_3$ separately. Similarly, in each of the 4 different vials 10 mg of Pluronic F127 was dissolved in 1 mL $CHCl_3$ separately. Subsequently, each of the probe solutions was mixed with each of the Pluronic F127 solutions and sonicated for 10 min to prepare a homogeneous solution. The $CHCl_3$ was then fully evaporated from each of the four mixtures carefully using the rotary vapour instrument. 1 mL of water was added to each of the samples and all the 4 samples were then sonicated again for 10 min to dissolve the solid residues. For AIETP, AIETP C2 and AIETP C3 the solid residues dissolved frequently in aqueous solutions whereas that of the AIETP C1 remained very poorly soluble (**Scheme S2**, Supporting Information). The solutions were then filtered with 0.45 µm filter and used as nanoparticles (AIETP NPs, AIETP C1 NPs*, AIETP C2 NPs and AIETP C3 NPs) of the corresponding probes for studies. (*Due to very poor solubility, filtration with 0.45 µm filter resulted in the aqueous solution of AIETP C1 NPs* with negligible probe concentration).

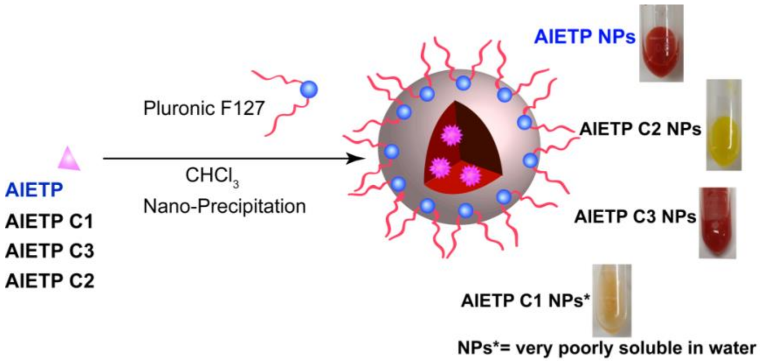

Scheme S2. Schematic illustration of the fabrication of nanoparticles.

Determination dye encapsulation efficiency of AIETP NPs: The amounts of the AIEgen (AIETP) effectively encapsulated into the AIETP NPs were determined from the absorption spectra. A calibration curve was obtained from the absorption spectra of AIETP with varying concentrations in aqueous solution. AIETP content in the AIETP NPs was then determined from the absorption spectra with reference to the calibration curve. The encapsulation efficiency **(**EE) of AIETP NPs was calculated from the ratio of the amount of the AIETP found in the AIETP NPs solution to the amount of AIETP used for AIETP NPs preparation.

Encapsulation efficiency= (Amount of AIETP found in the AIETP NPs solution/Amount of AIETP used for AIETP NPs preparation) ×100%= (15.8299/16) ×100%=99%

Important to note that;
Amount of AIETP used for AIETP NPs preparation: 1mg/mL= 16 µM
Amount of AIETP found in the AIETP NPs solution= 15.8299 µM (*determined from the calibration curve*; *Figure S25*)

Determination of concentration of AIETP NPs: AIETP NPs are stable and well-dispersible in aqueous solution. Hence, the density of the suspension of AIETP NPs could be estimated as 1 g cm^{-3}. Now, the average particle size of AIETP NPs is around 45 nm (radius) as determined by DLS and TEM analysis. Therefore, the concentration of AIETP NPs in aqueous solution (1 mg/mL) can be calculated using the following equation:

$$\text{Total number of AIETP NPs} = \frac{\text{Total volume of AIETP NPs}}{\text{Average volume of each AIETP NP}} = \frac{1\times10^{-3}\text{g}/1\text{g mL}^{-1}}{\frac{4}{3}\pi(45\times10^{-7})^3\text{mL}}$$

$=2.62\times10^{12}$

$$\text{AIETP NPs concentration}= \frac{2.62\times10^{12}/6.02\times10^{23}\ \text{mol}^{-1}}{1\times10^{-3}\ \text{L}} = 4.35\times10^{-9}\ \text{M} = 4.35\ \text{nM}$$

Fluorescence quantum yield measurement: The relative fluorescence quantum yield (η) of AIETP NPs in aqueous media was calculated using freshly prepared Rhodamine B as the standard (η = 0.7 in methanol). The η value of AIETP NPs was found to be 0.06 when calculated using the following equation:

$$\eta_1 = \eta_0 \frac{A_0 F_1 n_1}{A_1 F_0 n_0}$$

where, A is the absorbance at the excitation wavelength, F represents the integrated fluorescence intensity, n is the refractive index of the used solvent, and the subscripts 1 and 0 signify the experimental sample and the standard respectively.

Two-photon properties and the two-photon cross section: The aqueous solution of AIETP NPs, AIETP C2 NPs, AIETP C3 NPs and the AIETP C1 NPs* (*very poor solubility in water) were loaded in cell dishes and excited using femtosecond laser at different wavelengths from 820 to 1200 nm. The average two-photon emission intensities were calculated by Image J software for the four different samples.

To calculate the two-photon absorption cross section (σ_2) value of AIETP NPs the following equation was used -

$$(\sigma_2)_1 = (\sigma_2)_0 \frac{F_1 \eta_0 c_0 n_0}{F_0 \eta_1 c_1 n_1}$$

where F represents the two-photon emission intensity, η signifies the fluorescence quantum yield, c is the molar concentration of probe, n denotes the refractive index of the solvent, and the subscripts 1 and 0 represent the probe AIETP NPs and reference Rhodamine B respectively.

Cytotoxicity and cell imaging studies of AIETP NPs: Cytotoxicity studies were performed with the HeLa cells to evaluate the cell viability using Cell Counting Kit-8 (CCK-8) assay. Essentially, prior to CCK8 assay, 10^4 cells per well were seeded into a 96-well tissue culture plate and incubated in a humidified incubator for adherence. After 24 h of culturing, the medium in each well was replaced by 100 μL of fresh medium (RPMI-1640) containing varying concentrations (0-150 μM)) of AIETP NPs and incubated for a period of 24 h. The solution was filtrated subsequently using 0.22 μm sterile filter. The CCK-8 reagent, diluted by RPMI-1640 medium (10%) was added to each well after the removal of culture media and the plate was incubated for 0.5 h at 37°C. Later, the absorbance was measured at 450 nm on a plate reader (RT-6100, Rayto Life and Analytical Sciences Co., Ltd, Shenzhen, China). Each trial was performed with six parallel wells. Cell viability rate was determined using the formula VR = (As-Ab)/(Ac-Ab) × 100%, where As is the absorbance of the experimental group, Ac is the absorbance of the control group (no AIETP NPs), and Ab is the absorbance of the blank group (only CCK-8).

Cell imaging studies were performed with the HeLa cancer cells which were cultured in the RPMI-1640 medium. The culturing medium was supplemented with 10% (v/v) FBS (gibco) and 1 % antibiotics (10000 Units/mL penicillin and 10000 μg/mL streptomycin, gibco) under 5% CO_2 atmosphere and 95% humidity at 37 °C until the HeLa cells became approximately 80% confluent. The cells were then utilized for the imaging studies. The single photon and two photon cell imaging studies were then performed in a Nikon A1 Confocal Microscope. The cells were incubated with AIETP NPs in the culture medium for 24 h, followed by the removal of the medium and triple washing with phosphate buffered saline (PBS) before acquiring the cell imaging.

Intracellular ROS evaluation: Following the same procedure mentioned in the previous section HeLa cells were cultured until the cells were approximately 80% confluent. The cells were then incubated with AIETP NPs (5 μM) for 24 h. The cells were then washed with 1×PBS and incubated with DCFDA (20 μM) for 30 min. Again, the cells were washed with 1×PBS and subjected to 1040 nm two-photon light (8 mW) irradiation for 120 scans (a total of 12.68 min). Eventually, the cell imaging studies were performed with these cells with the Nikon A1 Confocal Microscope. Fluorescence emissions for DCFDA were collected in the range of 500–550 nm while the excitation was provided at 488 nm. On the other hand, 1040 nm two-photon

excitation was used for AIETP NPs and the emission was acquired at the range of 604–679 nm.

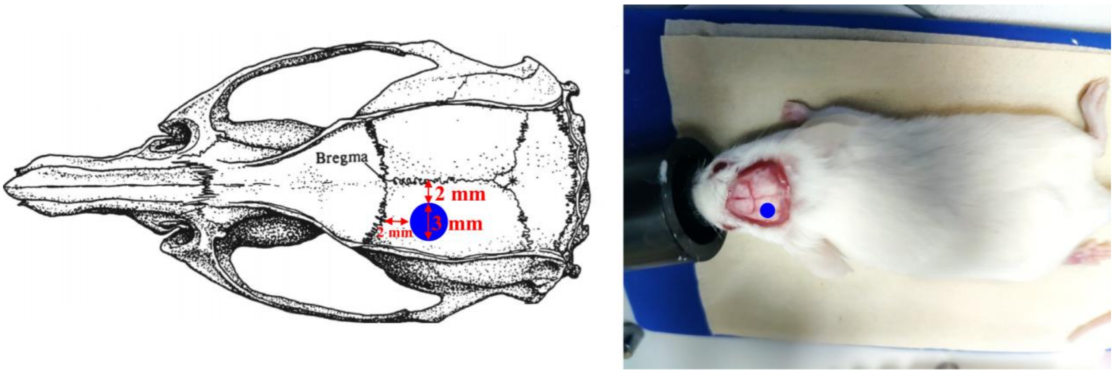

Scheme S3. Schematic illustration and the visual image of the exposed mouse brain taken under the experimental set up, highlighting the specific brain region which was captured to monitor the brain vasculature images.

***In vivo* brain imaging studies:** The animals, used for the *in vivo* studies were treated according to the protocols and the guidelines approved by Ethics committee of experimental animals, Medical department, Shenzhen University. The animals were prepared for the experiments following the standard procedure. Balb/c mice (male, 7-9 weeks old), provided by Guangdong Medical Laboratory Animal Centre were subjected to the *in vivo* two-photon fluorescence imaging experiment. In brief, the mouse was anesthetized first with a gaseous anaesthesia system (R500IP, RWD). A heating blanket was utilized to maintain the body temperature of mouse at 36 °C, and the head hair was removed and skin incision was performed to expose the skull. Thereafter, the head of a mouse was immobilized on a stereotactic stage with a heating blanket, a home-made titanium alloy piece with threaded holes was glued to the skull using dental cement. A small piece of skull bone with diameter around 3 mm was excised by a dental drill. The schematic illustration and the visual image of the exposed mouse brain under the experimental set up has been depicted in Scheme S3 (Supporting Information) before it was subjected to the *in vivo* imaging study. The aqueous solution (200 µL) of the prepared AIETP NPs (4.35×10^{-9} M) was administered through retro-orbital injection into the mouse vein. Femtosecond laser was focused on the surface of the brain through a 20× water immersion objective and z-stack imaging was performed with 5 µm step. The in vivo two-photon fluorescence imaging studies were then performed by setting the two-photon excitation laser at 1040 nm (NIR-II) wavelength.

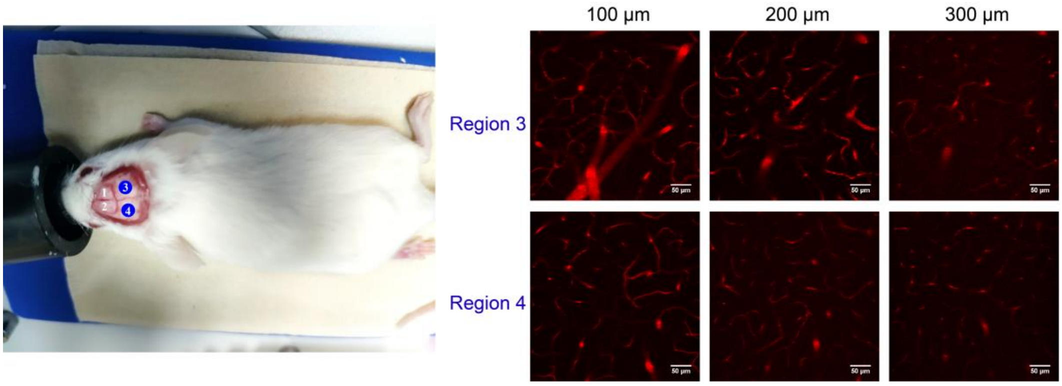

Scheme S4. Brain vasculatures in both the region 3 and 4 were successfully labeled by AIETP NPs, indicating towards the whole brain labelling aptitude of AIETP NPs.

Selecting the area for *in vivo* brain imaging: Since the positions of region 1 and 2 of the skull (Scheme S4, Supporting Information) are too small to meet the requirements of the cranial window of 3 mm; region 3 or 4 can only be chosen for imaging experiment. Before removing the skull bone, few drops of saline were poured onto the brain to make it translucent, so that the distribution of blood vessels on region 3 and 4 can be observed. Region 3, with fewer thick blood vessels, was finally selected for the extensive *in vivo* brain imaging studies, since the thick blood vessels in region 4 might interfere with the signals from the tiny deep blood vessels (Scheme S5, Supporting Information). Craniotomies were performed centered at 2 mm posterior and 2 mm lateral to the bregma point.
However, both the region 3 and 4 can be imaged using AIETP NPs, which was confirmed by *in vivo* imaging experiment (Scheme S4, Supporting Information) confirming the whole brain labelling aptitude of AIETP NPs.

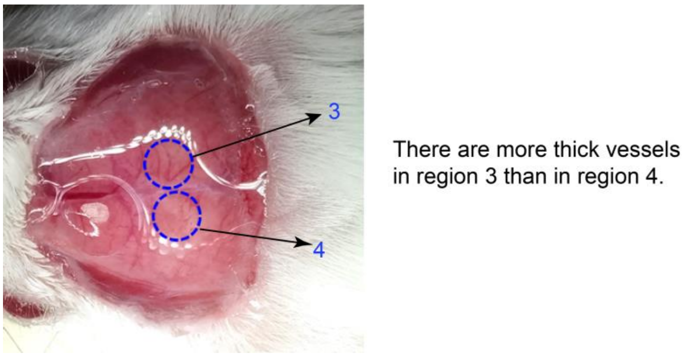

Scheme S5. Reason for choosing region 3 for extensive deep brain vasculature imaging using AIETP NPs.

***In vivo* toxicity:** For *in vivo* histological examination, Balb/c mice were intravenously injected with 200 μL of AIETP NPs (1 mg·mL^{-1} in 1×PBS), and major organs including brain, kidney, lung, spleen, liver, and heart were collected and stained with hematoxylin and eosin (H&E) after 6 days of administration. The control group was treated with only 200 μL of 1× PBS. The images were taken by an upright microscope (Leica EG11504) with a 200× objective.

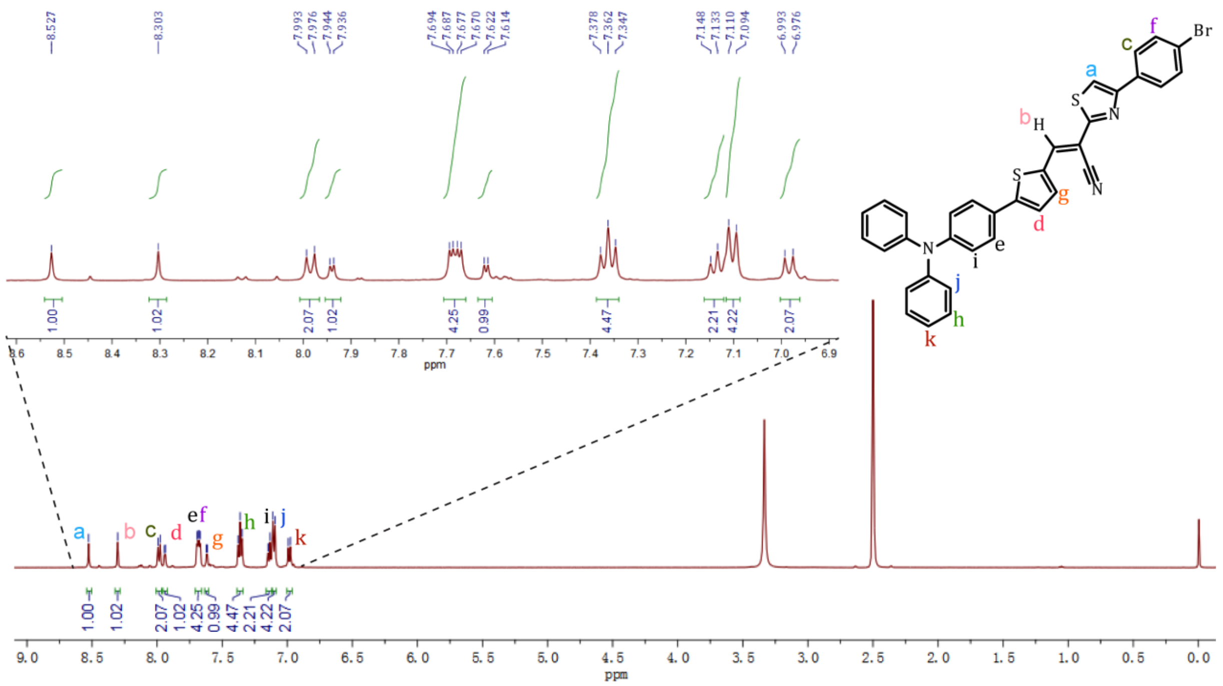

Figure S1. ^{1}H-NMR spectra of AIETP in DMSO-d_6.

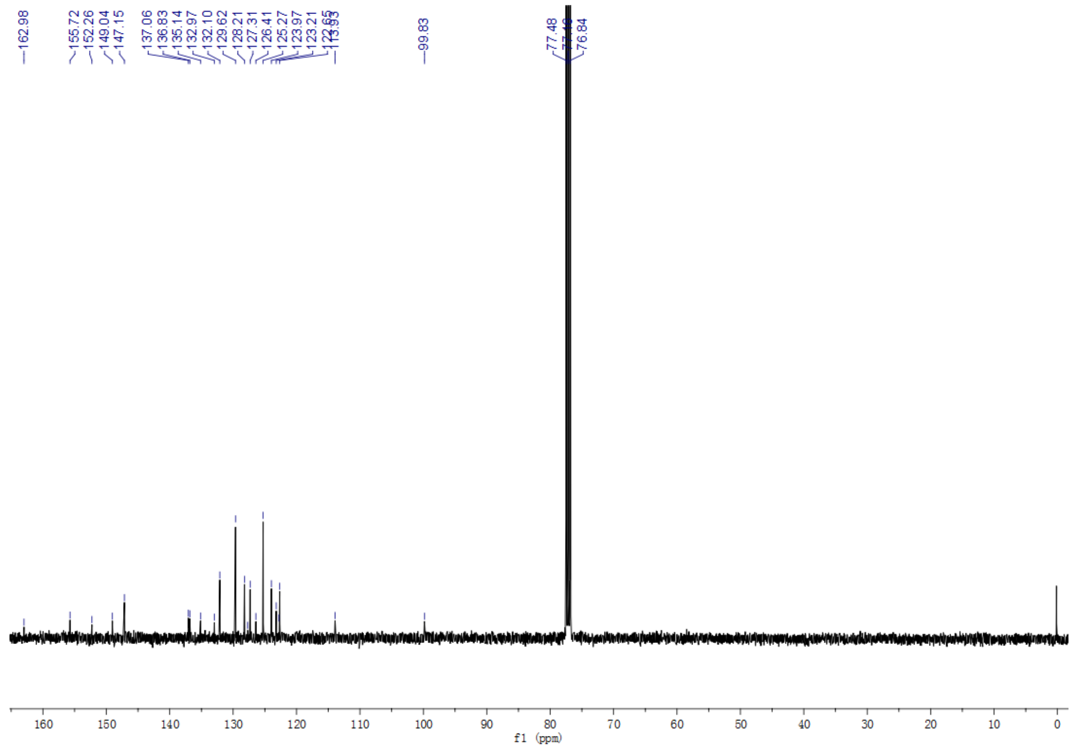

Figure S2. ^{13}C-NMR spectra of AIETP in $CDCl_3$.

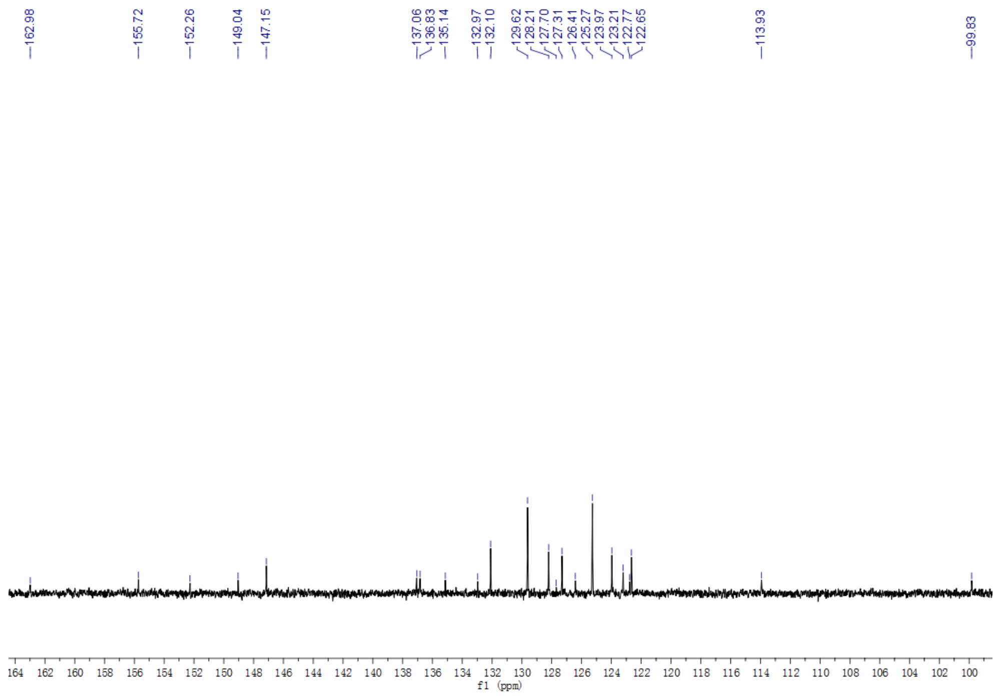

Figure S3. Expanded ^{13}C-NMR spectra of AIETP in $CDCl_3$.

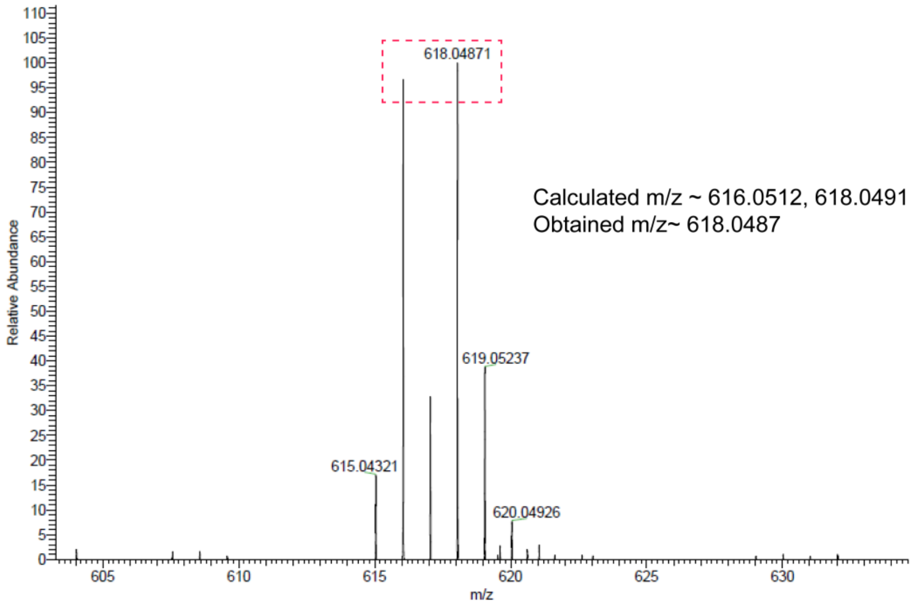

Figure S4. Mass spectrum of AIETP.

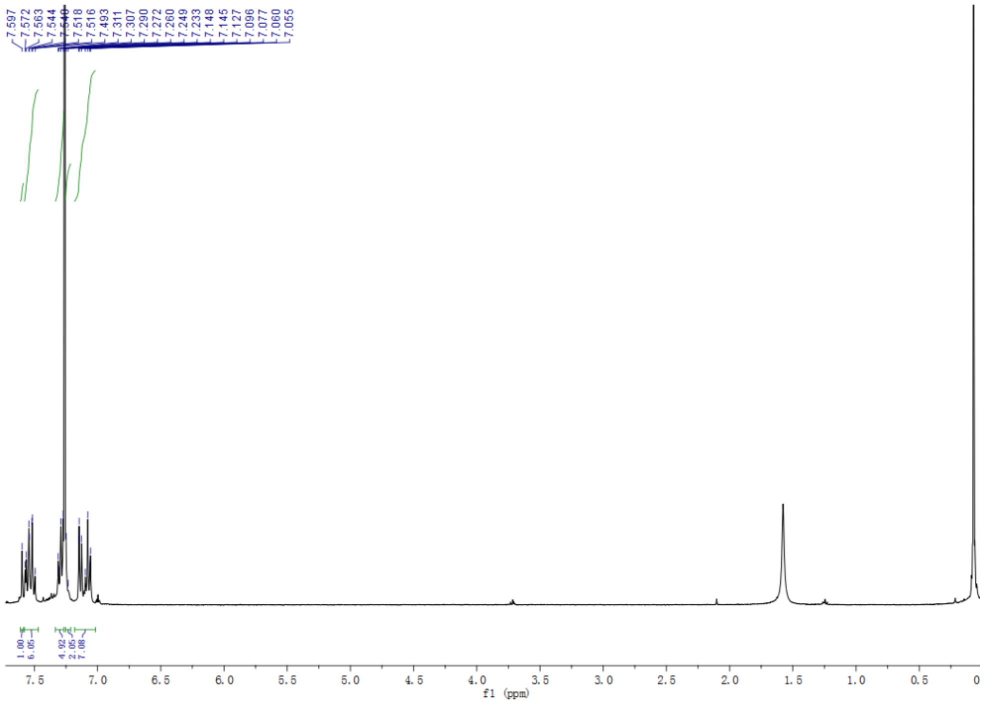

Figure S5. ^{1}H-NMR spectra of AIETP C1 in $CDCl_3$.

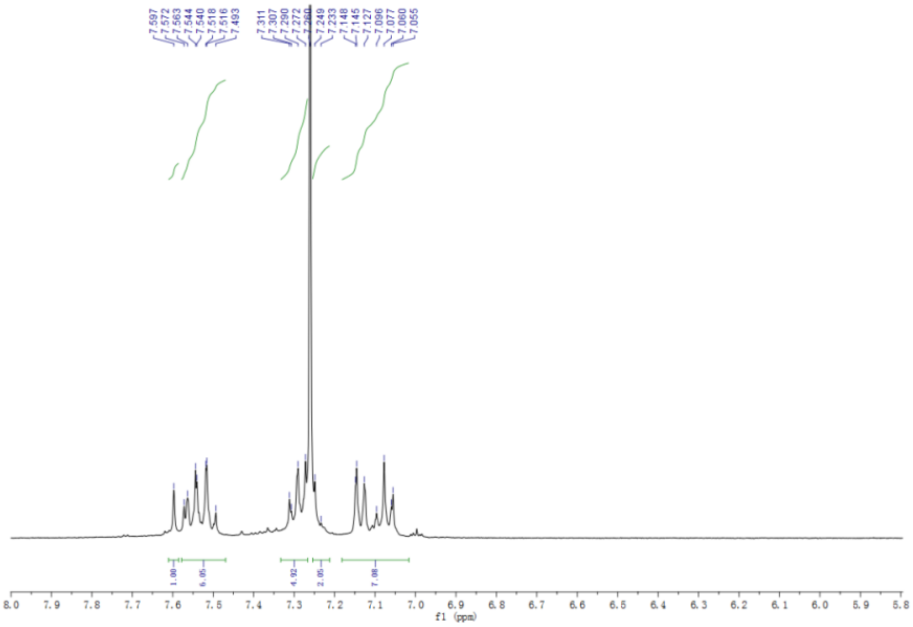

Figure S6. Expanded (aromatic region) ^{1}H-NMR spectra of AIETP C1 in $CDCl_3$.

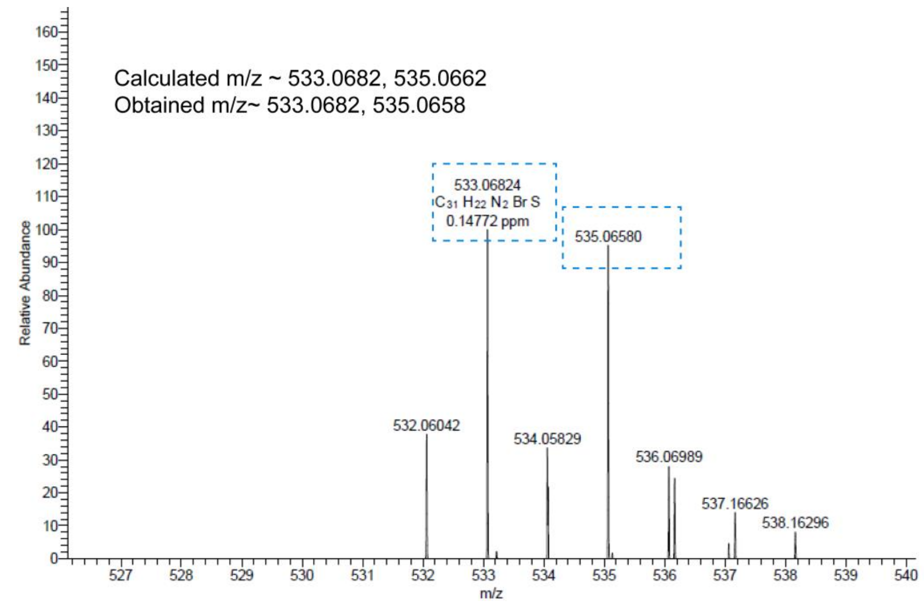

Figure S7. Mass spectrum of AIETP C1.

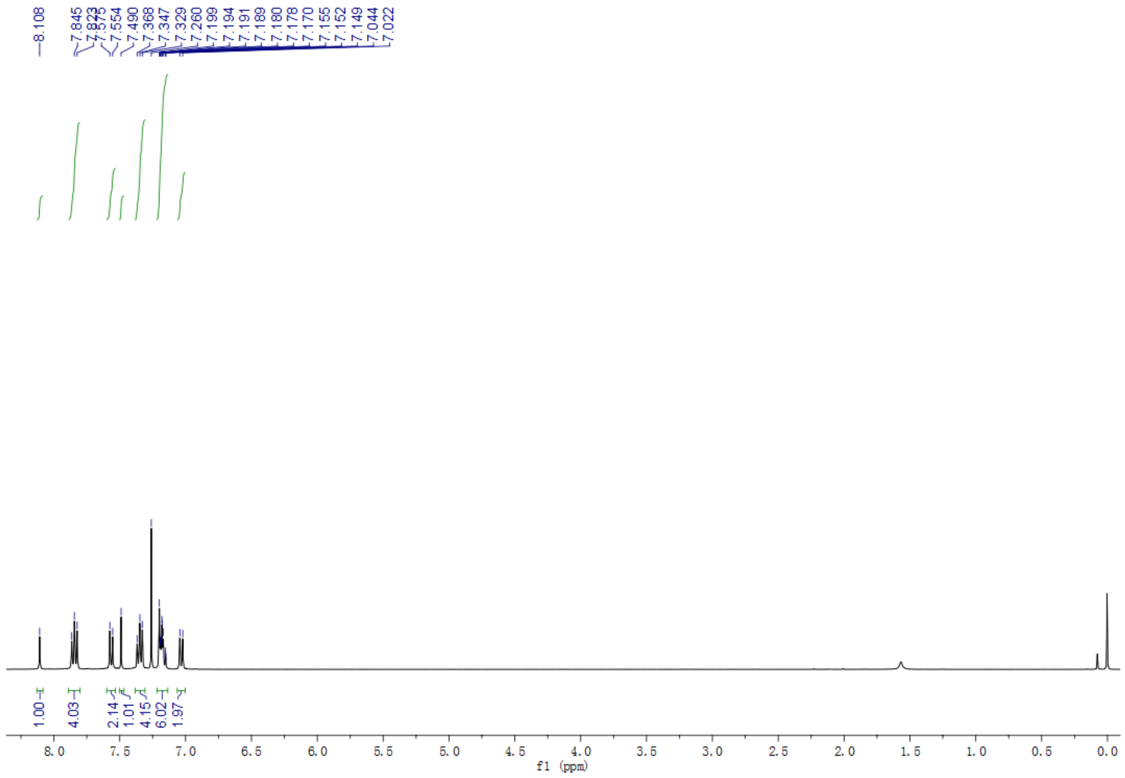

Figure S8. ^{1}H-NMR spectra of AIETP C2 in $CDCl_3$.

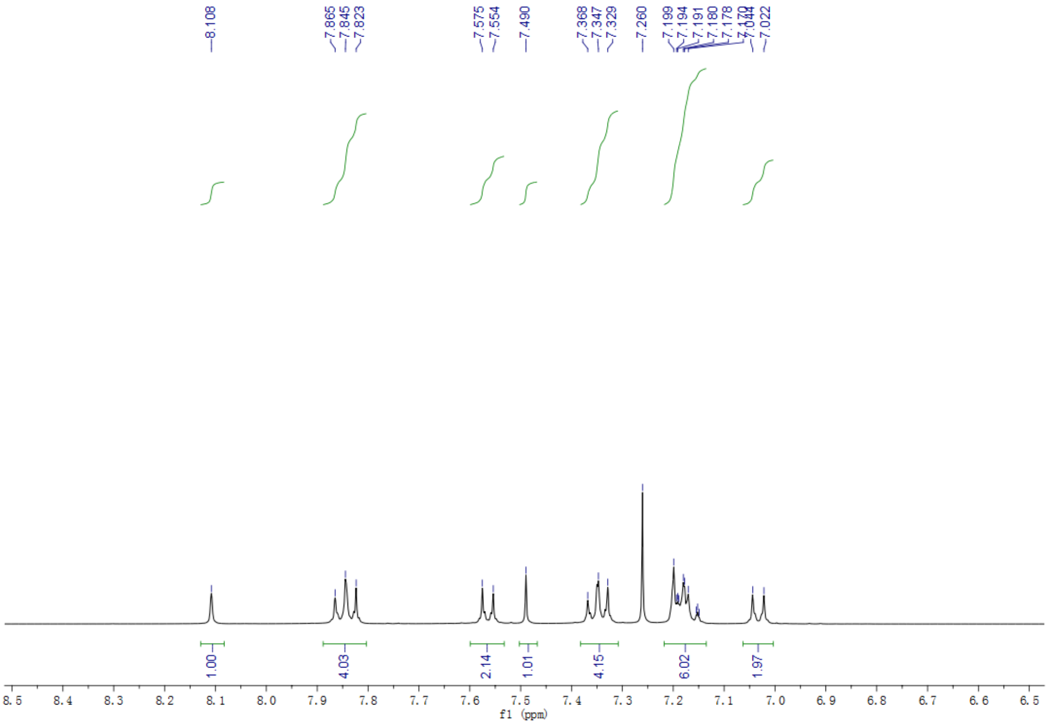

Figure S9. Expanded (aromatic region) ^{1}H-NMR spectra of AIETP C2 in $CDCl_3$.

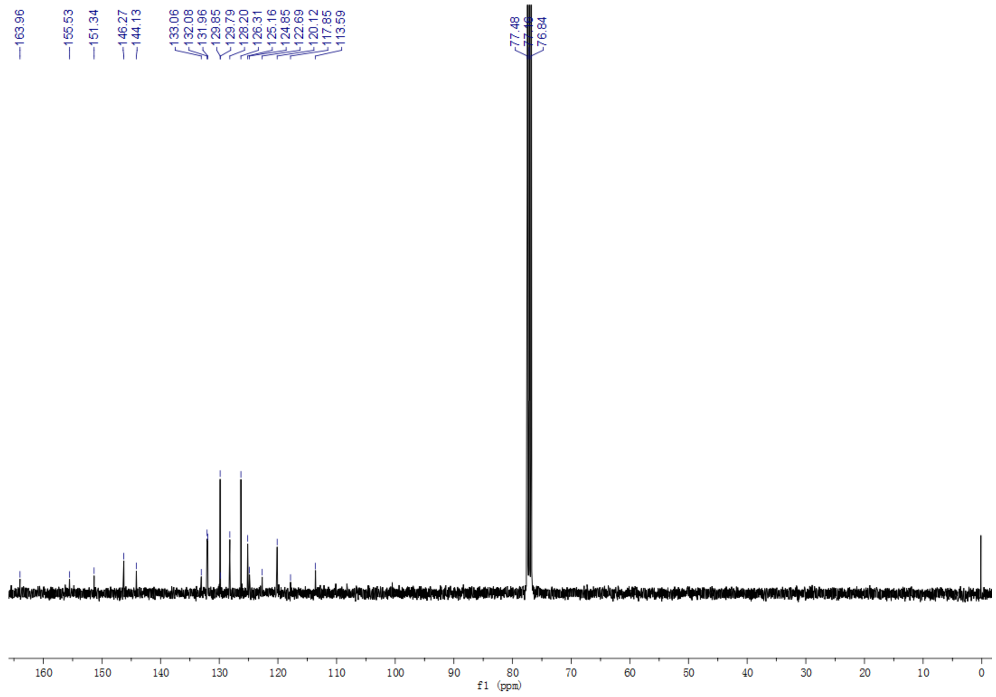

Figure S10. ^{13}C-NMR spectra of AIETP C2 in $CDCl_3$.

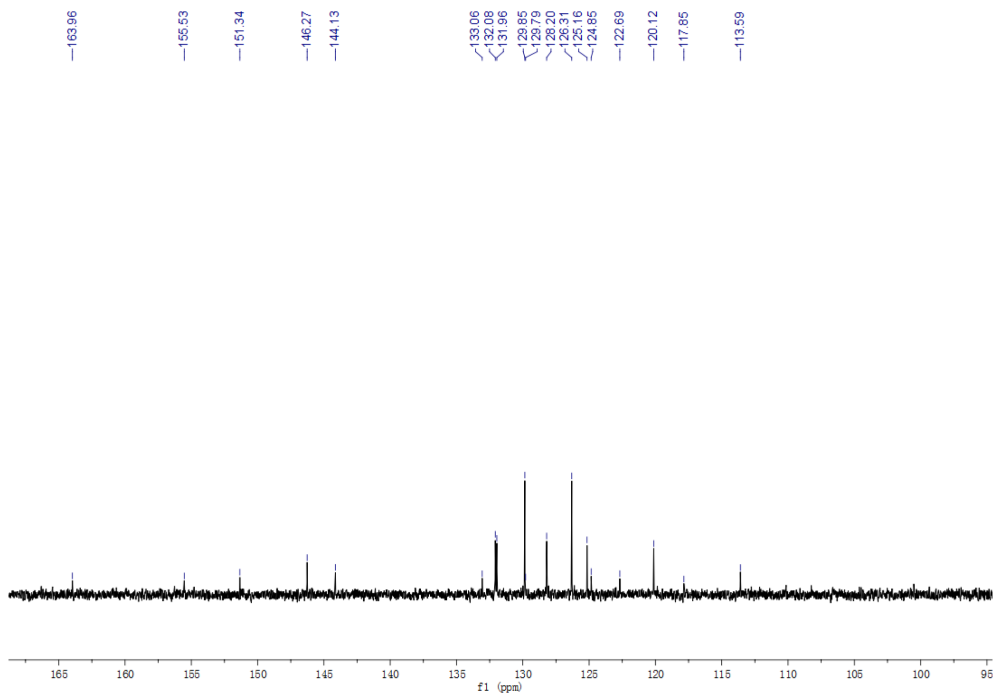

Figure S11. Expanded ^{13}C-NMR spectra of AIETP C2 in $CDCl_3$.

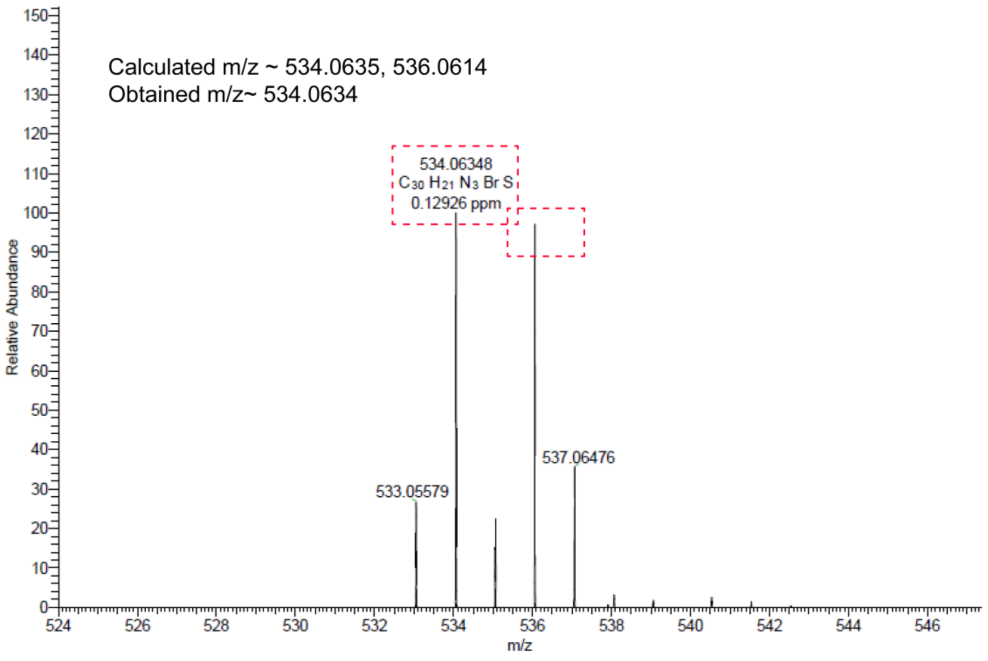

Figure S12. Mass spectrum of AIETP C2.

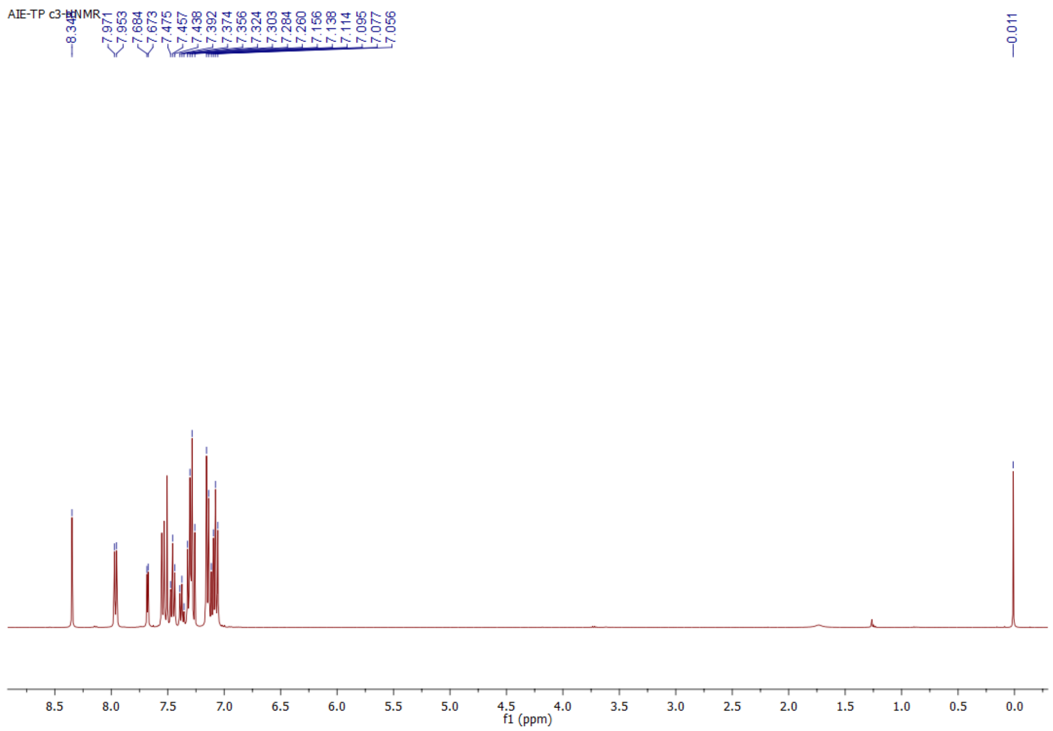

Figure S13. ^{1}H-NMR spectra of AIETP C3 in $CDCl_3$.

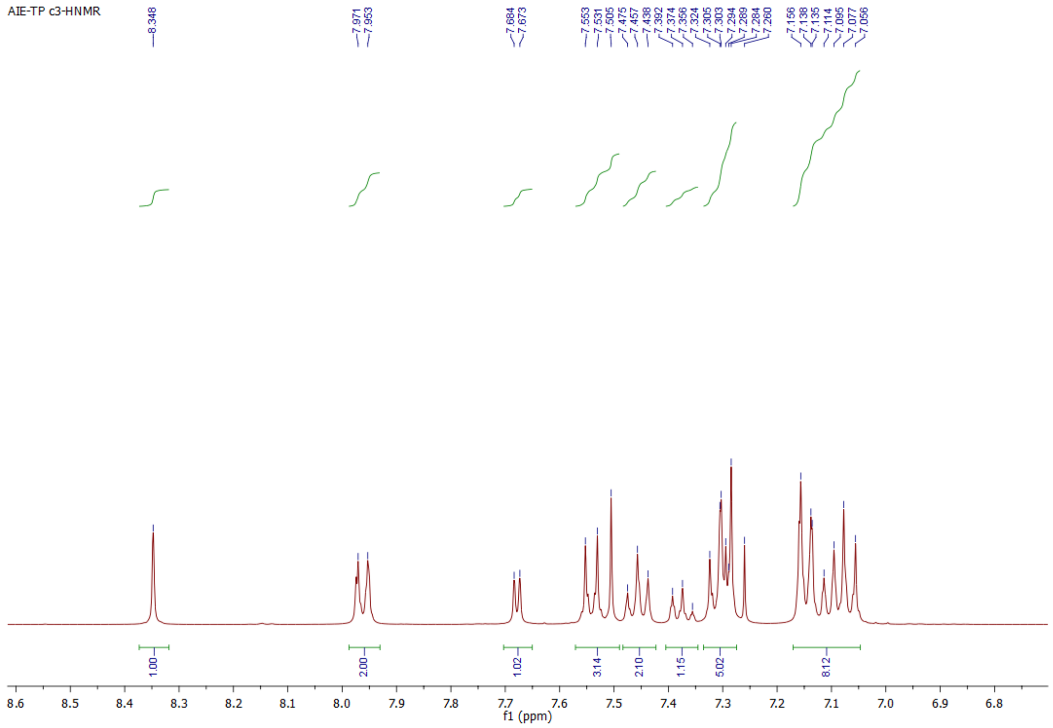

Figure S14. Expanded (aromatic region) ^{1}H-NMR spectra of AIETP C3 in $CDCl_3$.

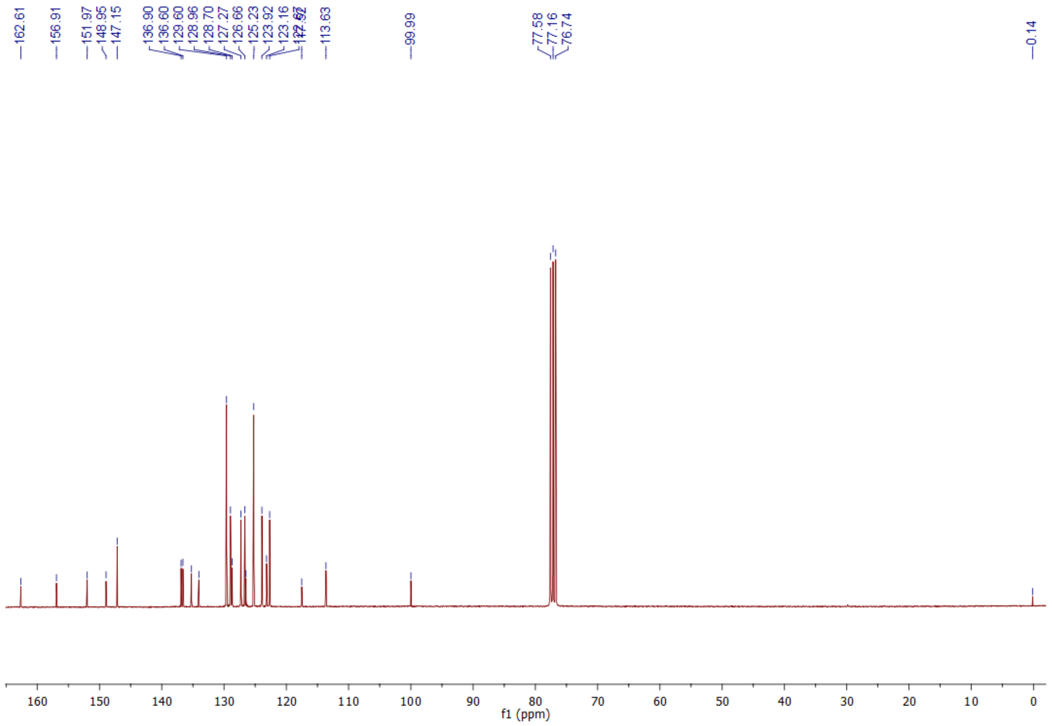

Figure S15. ^{13}C-NMR spectra of AIETP C3 in $CDCl_3$.

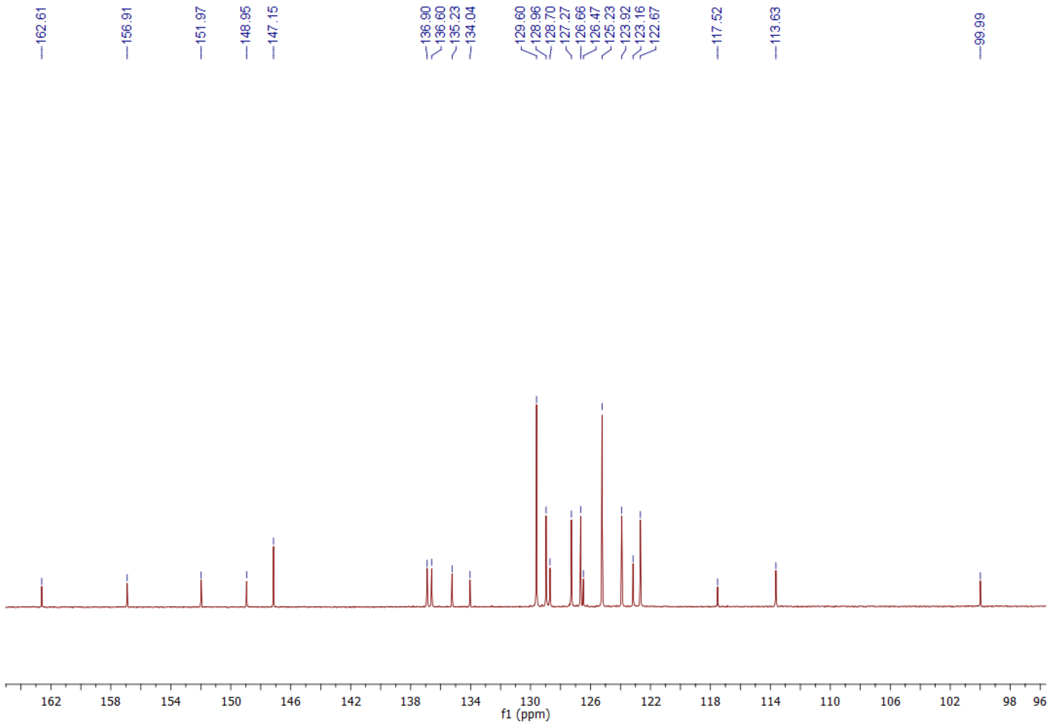

Figure S16. Expanded ^{13}C-NMR spectra of AIETP C3 in $CDCl_3$.

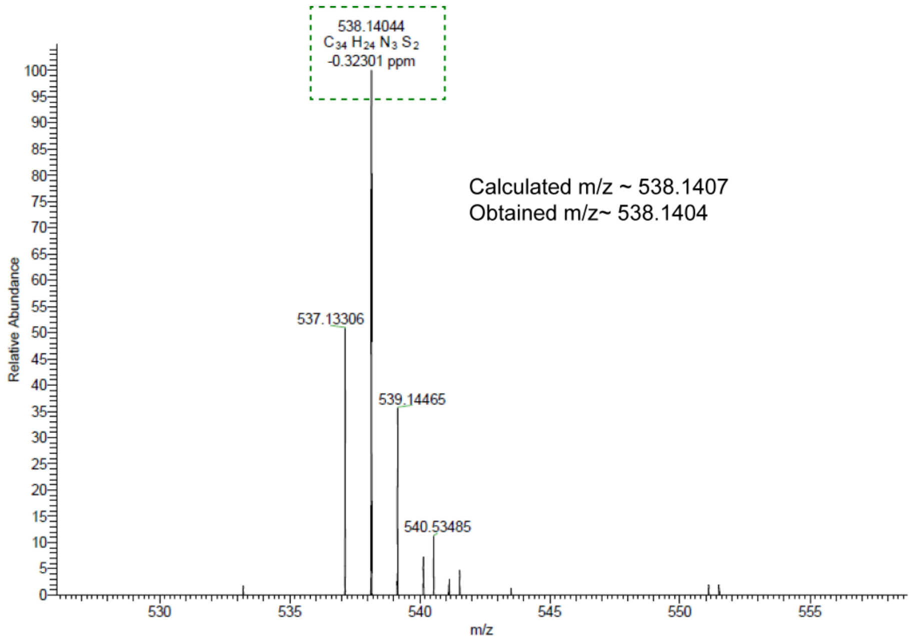

Figure S17. Mass spectrum of AIETP C3.

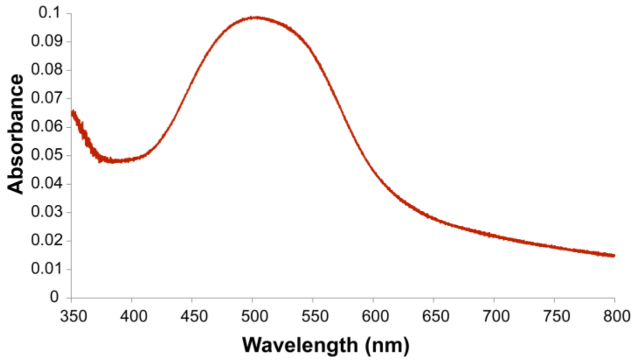

Figure S18. UV-Visible spectra of AIETP (10µM) in aqueous PBS medium**.**

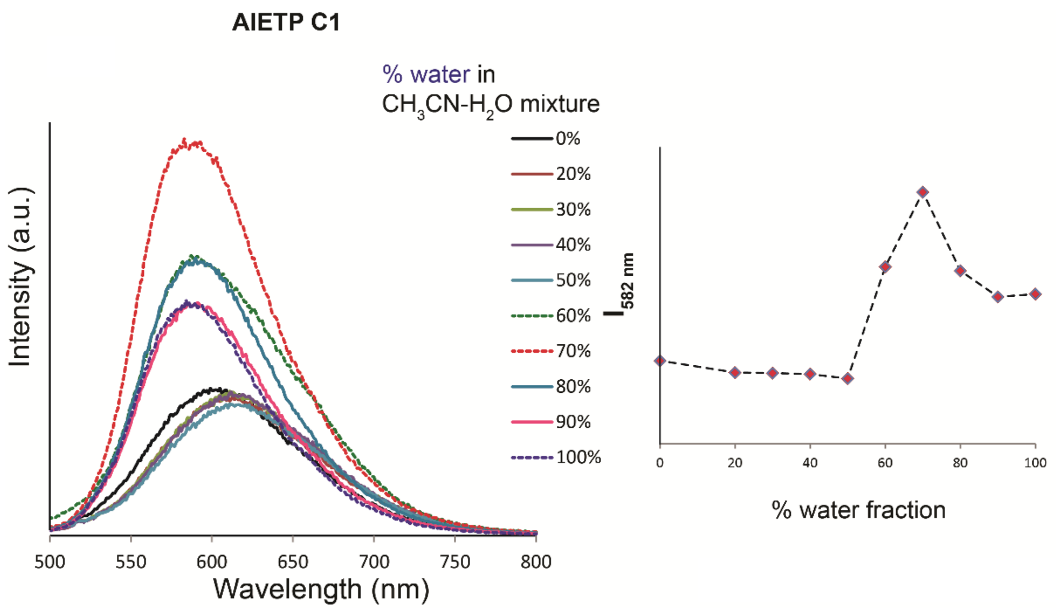

Figure S19. Emission spectra of AIETP C1 (10 µM) upon changing the water fraction of CH_3CN-water mixed solvent; λ_{ex} = 480 nm. Inset: the changes in the emission intensity at 582 nm with different water fractions.

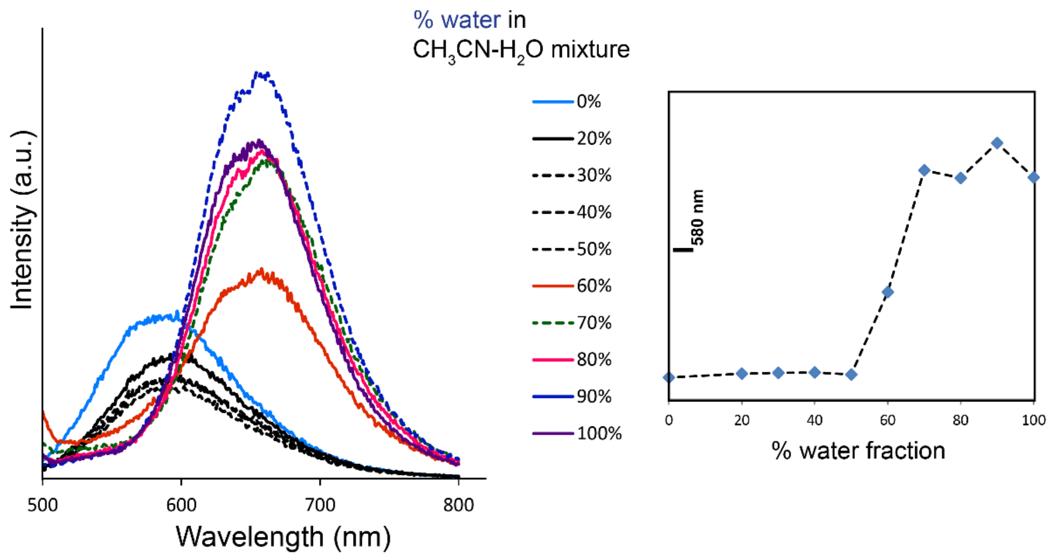

Figure S20. Emission spectra of AIETP C2 (10 µM) upon changing the water fraction of CH_3CN-water mixed solvent; λ_{ex} = 480 nm. Inset: the changes in the emission intensities (650/580 nm) at different water fractions.

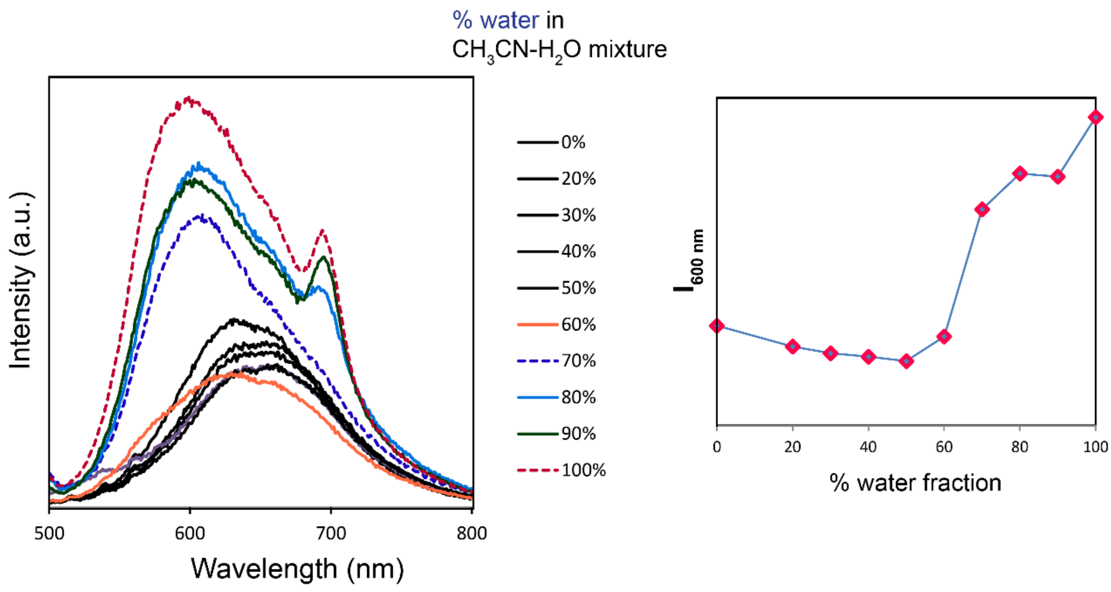

Figure S21. Emission spectra of AIETP C3 (10 µM) upon changing the water fraction of CH_3CN-water mixed solvent; λ_{ex} = 480 nm. Inset: the changes in the emission intensity at 600 nm with different water fractions.

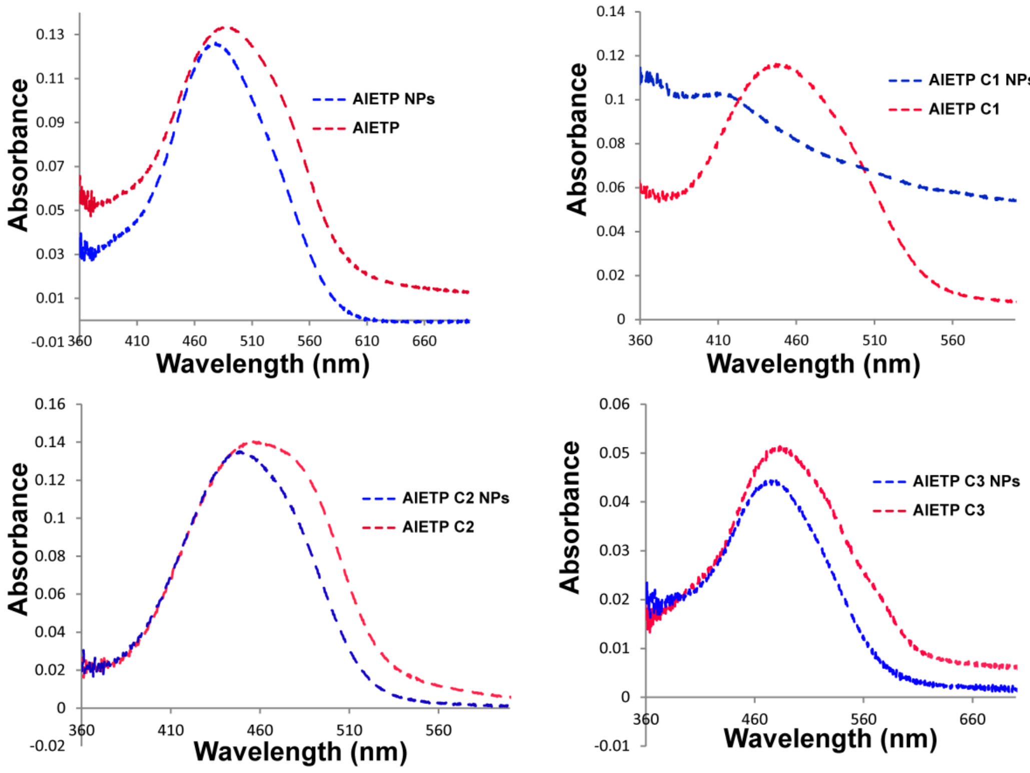

N.B.: For AIETP, AIETP C1, AIETP C2 and AIETP C3; UV-Vis spectra were recorded in aqueous medium containing 0.5% DMSO.

Figure S22. Changes in the UV-Vis spectra in aqueous media upon formation of nanoparticles.

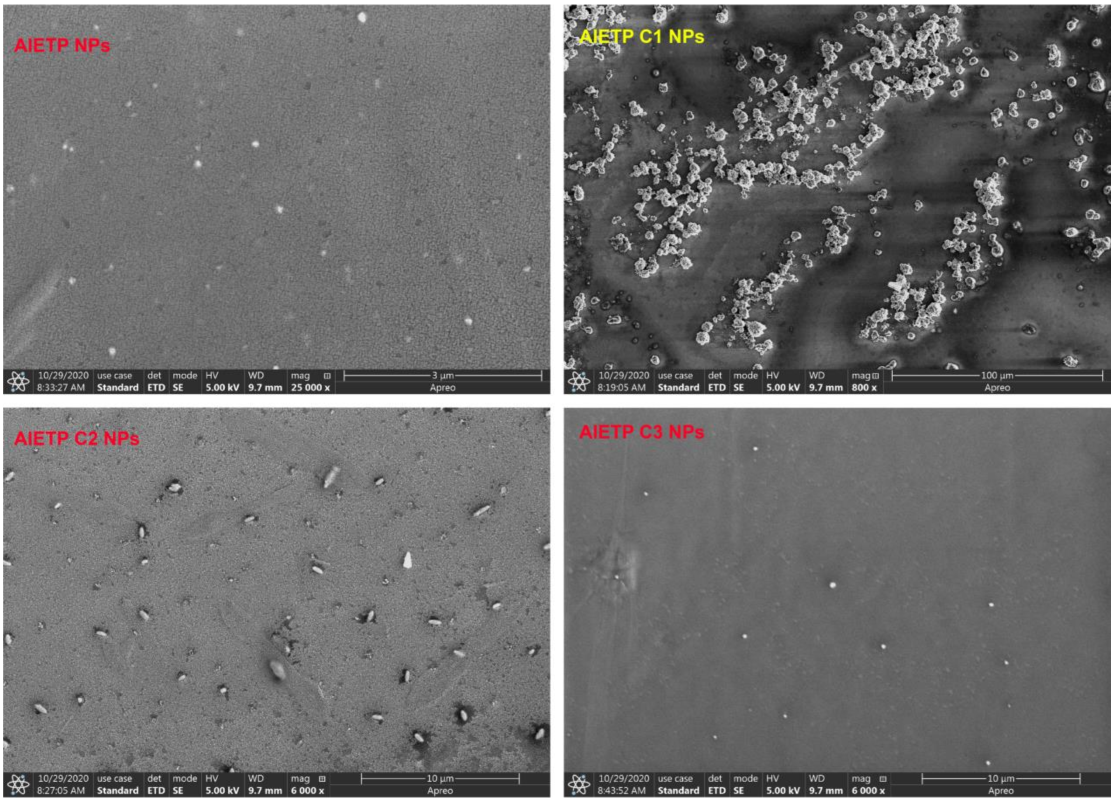

Figure S23. SEM images of the AIETP NPs, AIETP C1 NPs*, AIETP C2 NPs and AIETP C2 NPs. (AIETP C1 NPs*: very poor dispersibility in water).

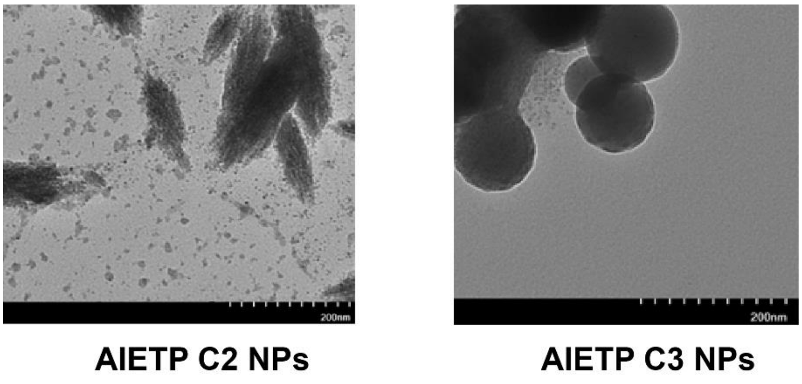

Figure S24. Characterization of the AIETP C2 NPs and AIETP C3 NPs via TEM analysis (scale bar 200 nm).

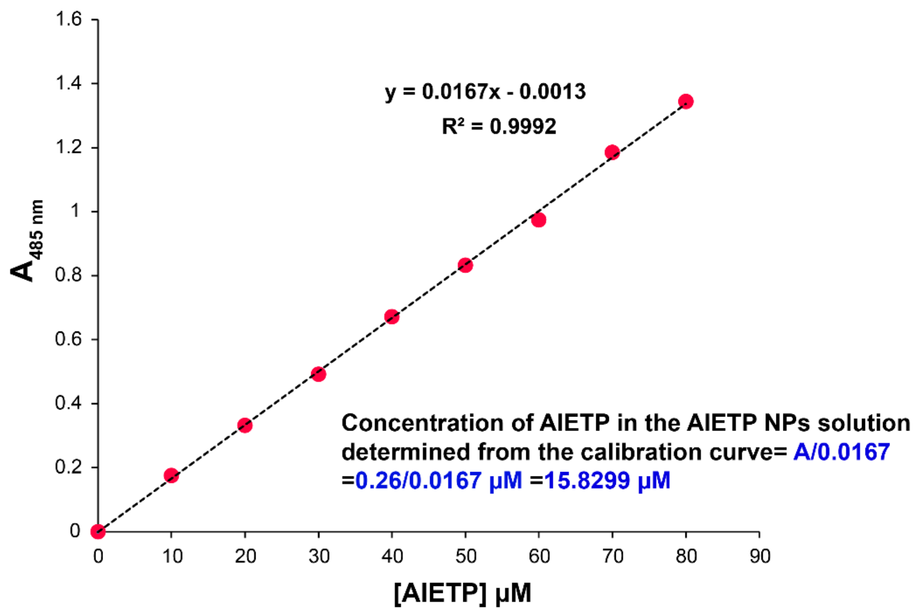

Figure S25. Determination of the concentration from absorption calibration curve of AIETP in aqueous medium.

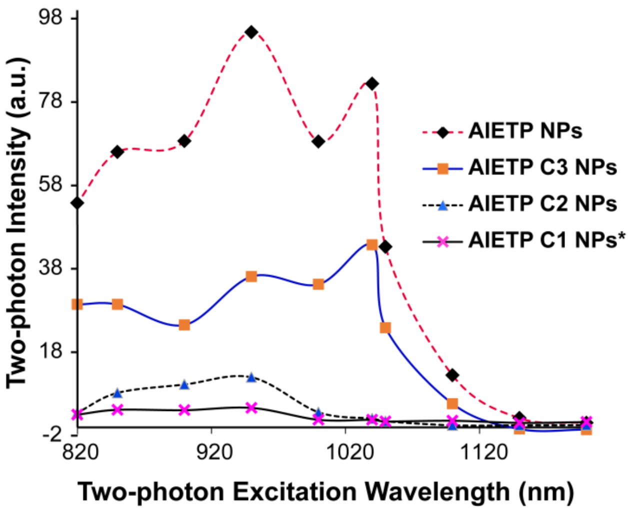

Figure S26. Two-photon fluorescence intensities upon changing two-photon excitation (820-1200 nm).

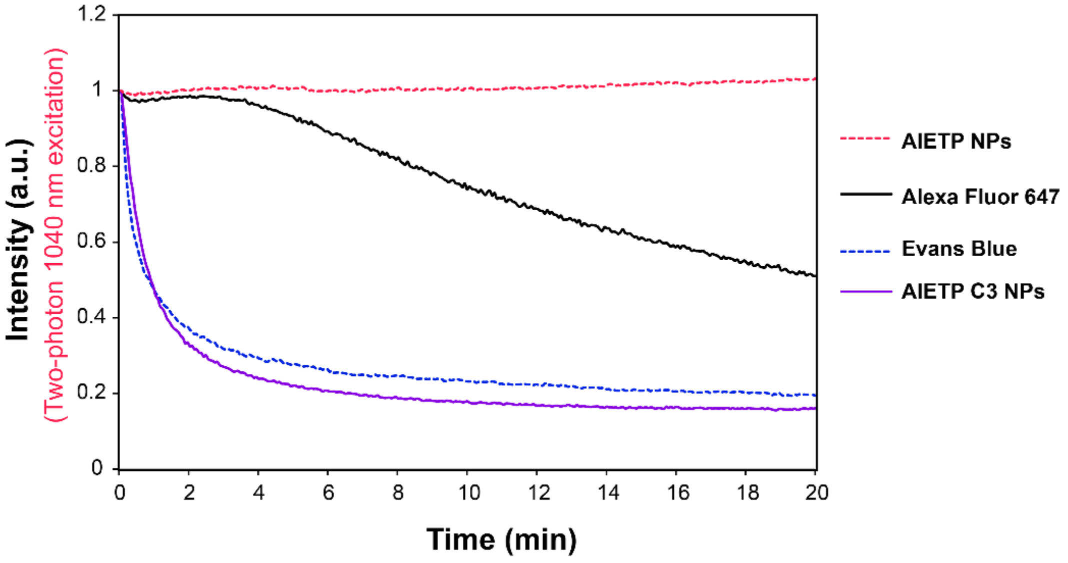

Figure S27. Variation in two-photon fluorescence intensities with time upon continuous two-photon excitation light irradiation at 1040 nm for AIETP NPs, AIETP C3 NPs, Evans blue and Alexa 647.

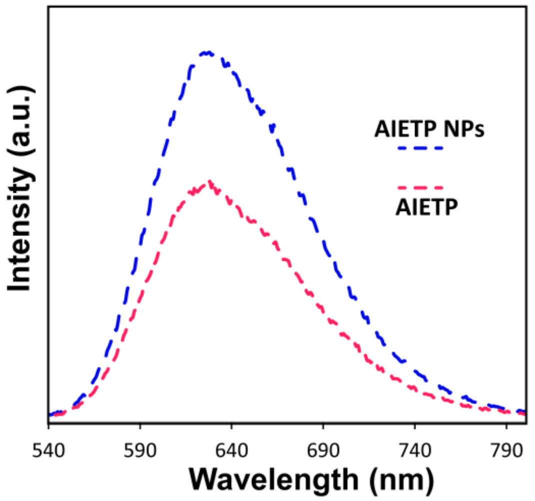

Figure S28. Change in fluorescence spectra of AIETP upon nanoparticle fabrication.

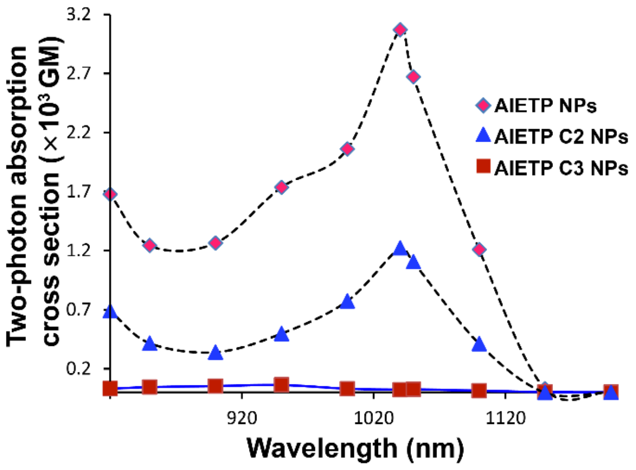

Figure S29. Two-photon absorption cross section of AIETP NPs, AIETP C2 NPs and AIETP C3 NPs at different two-photon excitations (820-1200 nm).

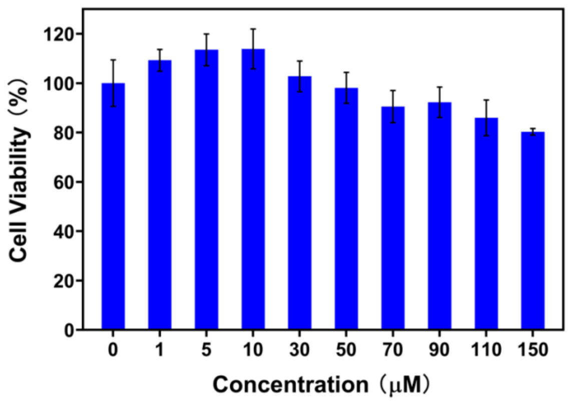

Figure S30. CCK-8 assay to determine the cytotoxic effect of AIETP NPs with varying concentrations on human cervical cancer (HeLa) cell line. The 'concentration' in the figure indicated to the concentration of the encapsulated AIETP in the AIETP NPs.

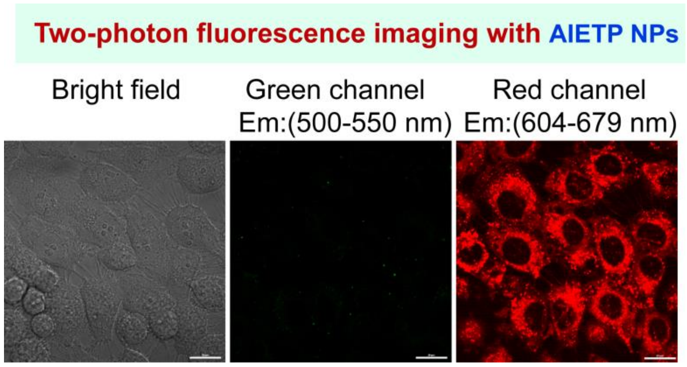

Figure S31. Two-photon fluorescence imaging of live HeLa cells with AIETP NPs; two-photon excitation: 1040 nm; Scale bar: 20 µm.

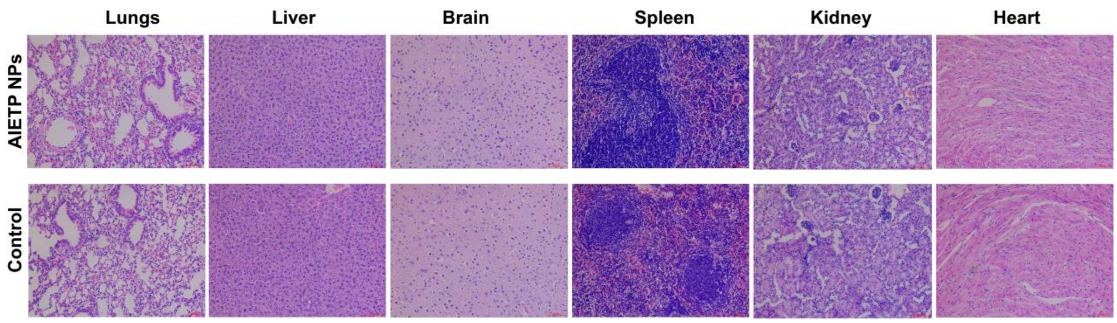

Figure S32. Histological examination of mice major organs (brain, kidney, lung, spleen, liver, and heart) stained with hematoxylin and eosin (H&E). Tissues were collected from mice after 6 days of the administration of 200 µL of AIETP NPs (1 mg·mL^{-1} in 1× PBS). The control mice were treated with 200 µL of 1× PBS only.

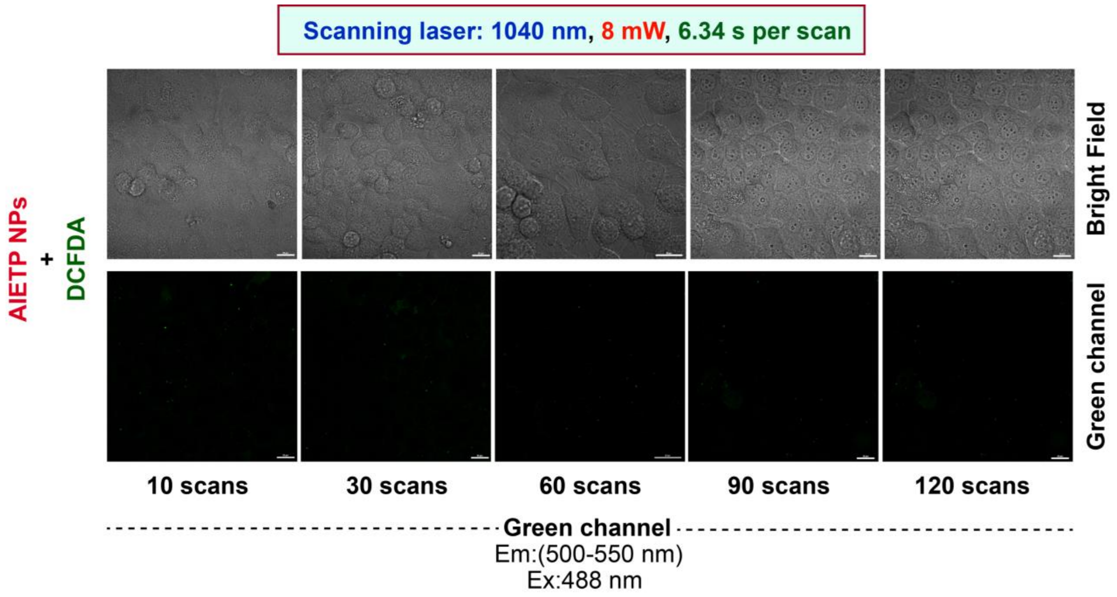

Figure S33. Evaluation of AIETP NPs induced intracellular ROS generation upon two-photon light irradiation. HeLa cells pre-incubated with AIETP NPs (5 µM) for 24 h followed by the incubation of DCFDA (20 µM) for 30 min and irradiated with 1040 nm two-photon laser (8 mW) for different scans were subjected to the confocal fluorescence imaging; Excitation: 488 nm and Emission: 500-550 nm. Scale bar: 20 µm.

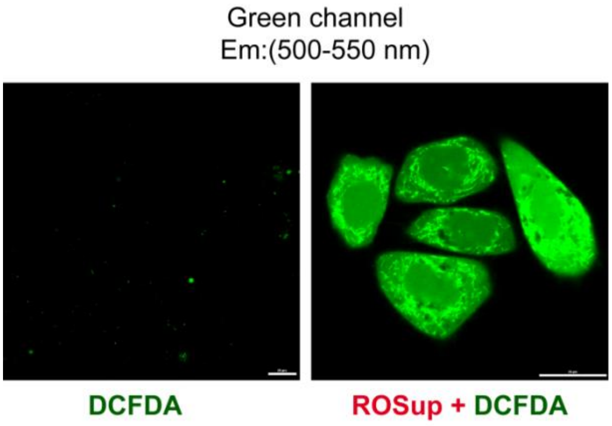

Figure S34. DCFDA (20 µM) labelled HeLa cells in absence and presence of ROS inducing reagent ROSup (0.05mg/mL). Scale bar: 20 µm.

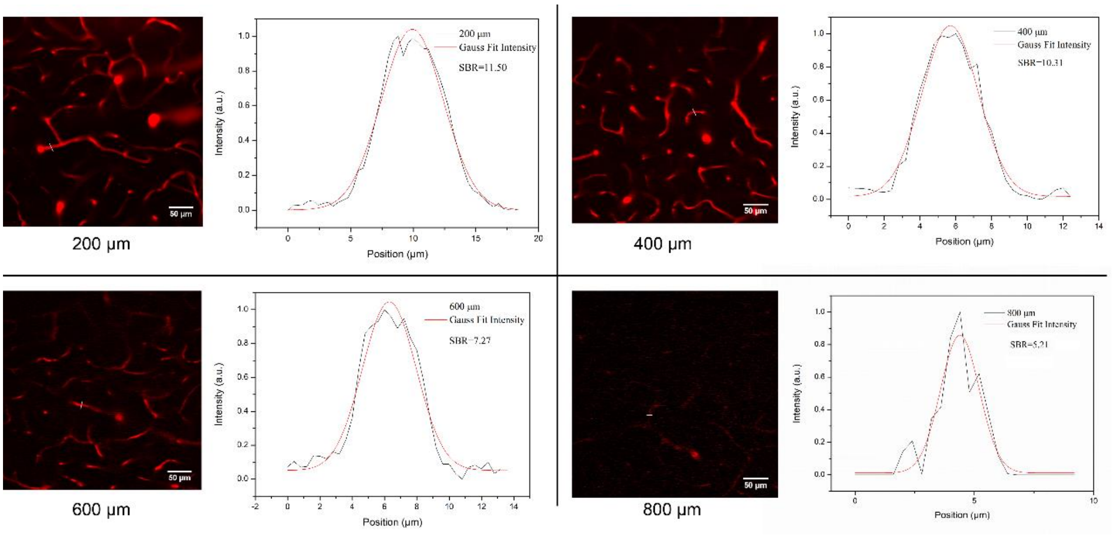

Figure S35. Signal-to background ratio (SBR) of the two-photon brain vasculature images at different depth measured from the two-photon fluorescence intensity line profiles across the blood vessels.

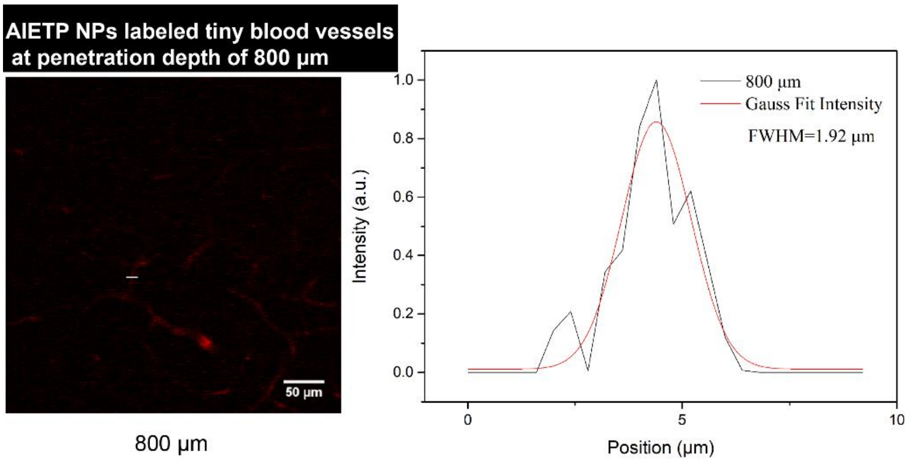

Figure S36. The line intensity profile of the blood capillary and the corresponding full width at half maximum (FWHM) Plot of the two-photon fluorescence image of mouse brain blood capillary at 800 μm depth to determine the resolution.

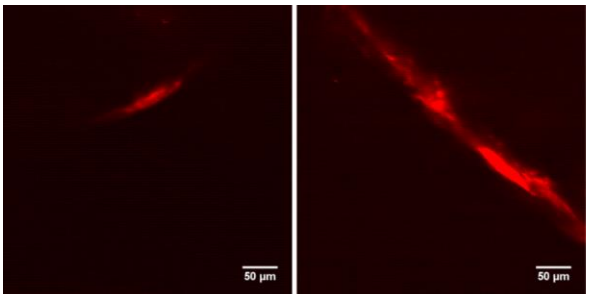

Figure S37. Magnified two-photon fluorescence image of the mouse brain blood vessels, 48 h after being labelled by AIETP NPs.

Table S1: Comparing the multi-photon fluorescent probes for brain vasculature imaging

**Fluorescent probe**	**Multi-photon excitation (nm)**	**Highest resolution (μm)**	**Largest Imaging depth (μm)**	**Reference**
P-dots NPs	1550 (3PF)	3.8	350	*Nano Research*, 2020, **13**, 2632–2640.
DCDPP-2TPA NPs	1550 (3PF)	2.4	300	*ACS Nano*, 2017, **11**, 10452–10461
DCzPDI-NPs	1550 (3PF)	2.31	450	*ACS Nano*, 2018, **12**, 9532–9540
AIE dots	1300 (2PF)	3.5	>840	*ACS Nano*, 2018, **12**, 7936–7945
PFBT NPs	810 (2PF)	–	500	*Chem. Mater.* 2014, 26, 1874–1880
Ant2-PHEA	800 (2PF)	–	600	*ACS Appl. Mater. Interfaces*, 2016, **8**, 17047–17059
PMMA NPs	780 (2PF)	–	800	*ACS Nano*, 2020, **14**, 9755–9770
BTPETQ dots	1200 (2PF)	2.5	924	*Adv. Mater.*, 2019, **31**, 1904447
CPdots1	1200 (2PF)	3.27	1010	*Adv. Funct. Mater.*, 2019, **29**, 1808365
TTS dots	900 (2PF)	4.0	350	*Chem. Sci.*, 2018, **9**, 2705–2710
DCCN	1040 (2PF)	2.7	800	*Adv Mater.* 2019, **31**,1904799
**AIETP NPs**	**1040 (2PF)**	**1.92**	**800**	**This report**
